# Analysis of inflammatory mediators in the vitreous humor of eyes with pan-uveitis according to aetiological classification

**DOI:** 10.1038/s41598-020-59666-0

**Published:** 2020-02-17

**Authors:** Hisako Fukunaga, Toshikatsu Kaburaki, Shintaro Shirahama, Rie Tanaka, Hiroshi Murata, Tomohito Sato, Masaru Takeuchi, Hideto Tozawa, Yoshihiro Urade, Mari Katsura, Mika Kobayashi, Youichiro Wada, Hirotsugu Soga, Hidetoshi Kawashima, Takahide Kohro, Makoto Aihara

**Affiliations:** 1 Department of Ophthalmology, University of Tokyo Graduate School of Medicine, 7-3-1 Hongo, Bunkyo-ku, Tokyo, 113-0033 Japan; 2 Department of Ophthalmology, Jichi Medical University Saitama Medical Center, Amanuma, Ohmiya-ku, Saitama, Saitama, 330-8503 Japan; 3 Department of Ophthalmology, National Defense Medical College, 3-2 Namiki, Tokorozawa, Saitama, 359-8513 Japan; 4 Isotope Science Center, The University of Tokyo, 2-11-16, Yayoi, Bunkyo-ku, Tokyo, 113-0032 Japan; 5 Department of Ophthalmology, Jichi Medical University, 3311-1 Yakushiji, Shimotsuke, Tochigi, 329-0498 Japan; 6 Department of Medical Informatics, Jichi Medical University, 3311-1 Yakushiji, Shimotsuke, Tochigi, 329-0498 Japan

**Keywords:** Haematological cancer, Eye diseases, Immunological disorders, Infectious diseases

## Abstract

Treatment of uveitis is complicated because of its multiple aetiologies and elevation of various inflammatory mediators. To determine the mediators that are elevated in the vitreous humor according to the aetiology of the uveitis, we examined the concentrations of 21 inflammatory cytokines, 7 chemokines, and 5 colony-stimulating/growth factors in vitreous samples from 57 eyes with uveitis associated with intraocular lymphoma (IOL, n = 13), sarcoidosis (n = 15), acute retinal necrosis (ARN, n = 13), or bacterial endophthalmitis (BE, n = 16). Samples from eyes with idiopathic epiretinal membrane (n = 15), which is not associated with uveitis, were examined as controls. Heat map analysis demonstrated that the patterns of inflammatory mediators in the vitreous humor in eyes with uveitis were disease-specific. Pairwise comparisons between the 5 diseases showed specific elevation of interferon-α2 in ARN and interleukin (IL)-6, IL-17A, and granulocyte-colony stimulating factor in BE. Pairwise comparisons between IOL, ARN, and BE revealed that levels of IL-10 in IOL, RANTES (regulated on activation, normal T cell expressed and secreted) in ARN, and IL-22 in BE were significantly higher than those in the other 2 types of uveitis. These mediators are likely to be involved in the immunopathology of specific types of uveitis and may be useful biomarkers.

## Introduction

Uveitis affects more than 2 million people worldwide^1^, is a leading cause of visual impairment in the working population, and accounts for 10% of the worldwide burden of blindness^2^. Uveitis is defined as intraocular inflammation involving the iris, ciliary body, and choroid and has more than 60 known aetiologies^3^. Depending on the aetiology, uveitis is classified clinically by the International Uveitis Study Group as infectious uveitis (IU), non-infectious uveitis (NIU), masquerade syndrome (malignant), or other (idiopathic)^4^. IU is caused by infectious agents, including viruses, bacteria, fungi, and protozoa. Acute retinal necrosis (ARN) is a representative type of IU and is a devastating necrotising retinitis caused by herpes simplex virus (HSV) or varicella zoster virus (VZV)^5^. Bacterial endophthalmitis (BE) is also a leading IU that causes severe necrotising retinitis and dense vitritis; it usually progresses rapidly and has a poor visual prognosis. On the other hand, intraocular lymphoma (IOL), also known as vitreoretinal lymphoma, is representative of masquerade syndrome, which is characterised by two ocular manifestations, i.e., vitreous cellular infiltration and subretinal tumor infiltration^6^. IOL is a life-threatening disease because of its high relapse rate in the central nervous system despite aggressive systemic chemotherapy and/or radiotherapy^7^, and its 5-year overall survival rate has been reported to be 61%^8^. Therefore, in clinical practice, early diagnosis of uveitis and appropriate aetiology-specific treatment is important. However, the diagnosis of uveitis is often challenging because there are many disease entities involved and their clinical features may be similar.

A diagnosis of uveitis can be expected in about 60–70% of cases using a systematic approach that includes history-taking, clinical examination, laboratory tests, and other investigations^9,10^. Tapping of aqueous humor or vitreous biopsy is sometimes needed to confirm the diagnosis, especially in cases that are suspicious for IU or masquerade syndrome. The samples obtained are used for laboratory tests, including polymerase chain reaction (PCR) to detect the DNA of microorganisms causing IU^11^, culture for bacteria or fungi, smears for cytology, the interleukin (IL)-10/IL6 ratio, and/or immunoglobulin heavy chain (IgH) rearrangement, which is needed for a diagnosis of IOL^7^.

Inflammatory cytokines and chemokines are elevated in the aqueous and vitreous fluid of eyes with uveitis, especially pan-uveitis, in which intraocular inflammation involves both the anterior chamber and posterior segment, and play a major role in the pathogenesis and persistence of intraocular inflammatory and degenerative diseases^12,13^. However, there have been no published comparisons of the levels of these mediators in the vitreous fluid of eyes with uveitis according to aetiology, e.g., IU, NIU, and masquerade syndrome. In this study, we determined the levels of 33 inflammatory mediators (Table 1) in vitreous samples from patients who were grouped according to aetiology of uveitis (IOL, sarcoidosis, ARN, or BE) and from controls with idiopathic epiretinal membrane (ERM, a disease that does not include uveitis). We then compared the levels of these inflammatory mediators between the eyes with uveitis according to aetiology and with those in the controls to identify the cytokines that are specifically elevated in each of four diseases that cause uveitis.

**Table 1:** Immune mediators analysed in the study.

Interleukins (ILs)	
	Interleukin-1 beta (IL-1β)
	Interleukin-1 receptor antagonist (IL-1ra)
	Interleukin-2 (IL-2)
	Interleukin-4 (IL-4)
	Interleukin-5 (IL-5)
	Interleukin-6 (IL-6)
	Interleukin-7 (IL-7)
	Interleukin-9 (IL-9)
	Interleukin-10 (IL-10)
	Interleukin-12 subunit p40 (IL-12(p40))
	Interleukin-12 subunit p70 (IL-12(p70))
	Interleukin-13 (IL-13)
	Interleukin-15 (IL-15)
	Interleukin-17A (IL-17A)
	Interleukin-22 (IL-22)
	Interleukin-26 (IL-26)
	Interleukin-27p28 (IL-27(p28))
Interferons (IFNs)	
	Interferon-alpha2 (IFN-α2)
	Interferon-beta (IFN-β)
	Interferon-gamma (IFN-γ)
Tumor necrosis factor (TNF)	
	Tumor necrosis factor-alpha (TNF-α)
Chemokines	
	Interleukin-8 (IL-8)
	Eotaxin (Eotaxin)
	Monocyte chemoattractant protein-1 (MCP-1)
	Macrophage inflammatory protein-1 alpha (MIP-1α)
	Macrophage inflammatory protein-1 beta (MIP-1β)
	Regulated on activation normal T cell expressed and secreted (RANTES)
	Interferon gamma-induced protein-10 (IP-10)
Colony-stimulating factors (CSFs)	
	Granulocyte-colony stimulating factor (G-CSF)
	Granulocyte macrophage-colony stimulating factor (GM-CSF)
Growth factors (GF)	
	Basic fibroblast growth factor (bFGF)
	Platelet-derived growth factor-BB (PDGF-BB)
	Vascular endothelial growth factor (VEGF)

## Results

Table 2 shows the characteristics of the 56 patients with uveitis and the 15 controls with ERM examined in this study. The mean age of the study population at the time of vitrectomy was 63.6 ± 13.8 years. The patients with ARN were relatively younger than those in the other study groups. The male to female ratio was higher in the idiopathic ERM, ARN, and BE groups and lower in the IOL and sarcoidosis groups. Best-corrected visual acuity before vitrectomy was worse in patients with BE. In the ARN group, PCR was positive for VZV-DNA in 12 cases (92.3%) and for HSV-DNA in 1 case (7.7%). In the BE group, cultures of vitreous specimens were positive in 6 cases (37.5%); the remaining 10 (62.5%) were negative but all were positive on PCR for the 16S bacterial ribosomal RNA gene^14^ and responded to antibiotic therapy. The patients’ clinical details at the time of sample collection are summarised in Supplementary Table S1. We collected samples from eyes with ARN or BE earlier in relation to the onset of symptoms than from those with IOL or sarcoidosis. The level of intraocular inflammation (anterior inflammation, presence or absence of hypopyon, and vitreous opacity) at the time of surgery was the most severe in eyes with BE. Retinitis at the time of surgery was more severe in eyes with ARN or BE (Supplementary Table S1).

**Table 2:** Patient demographics and ophthalmic characteristics.

Diseases	ERM	IOL	sarcoidosis	ARN	BE
Category	control	tumor	autoimmunity	virus infection	bacterial infection
Number of Patients	15	12	15	13	16
Ages (yrs)	65.43 ± 11.4133	68.92 ± 14.18	67.73 ± 11.34	51.15 ± 13.68	64.94 ± 11.45
Sex (male:female)	11:4	4:8	5:10	9:4	11:5
**Visual acuity before vitrectomy**					
<2/200	0	0	0	1	10
2/200≤ and <20/200	1	0	1	4	4
2/20≤ and <20/20	14	8	12	6	2
20/20≤	0	5	2	2	0
**Causative infectious agents**					
Virus	—	—	—		
HSV	—	—	—	1	
VZV	—	—	—	12	
cytomegalovirus	—	—	—	0	
Bacteria	—	—	—		
*Staphyococcus aureus*	—	—	—		2
coagulase-negative *Staphylococci*					1
*Streptococcus oralis*	—	—	—		1
*α-Streptococci*	—	—	—		1
*Klebsiella pneumoniae*					
	—	—	—		1
Positive PCR*	—	—	—		10

^*^Positive PCR for bacterial ribosomal RNA S16. ARN, acute retinal necrosis; BE, bacterial endophthalmitis; ERM, idiopathic epiretinal membrane; HSV, herpes simplex virus; IOL, intraocular lymphoma; PCR, polymerase chain reaction; VZV, varicella zoster virus.

**Figure 1:**
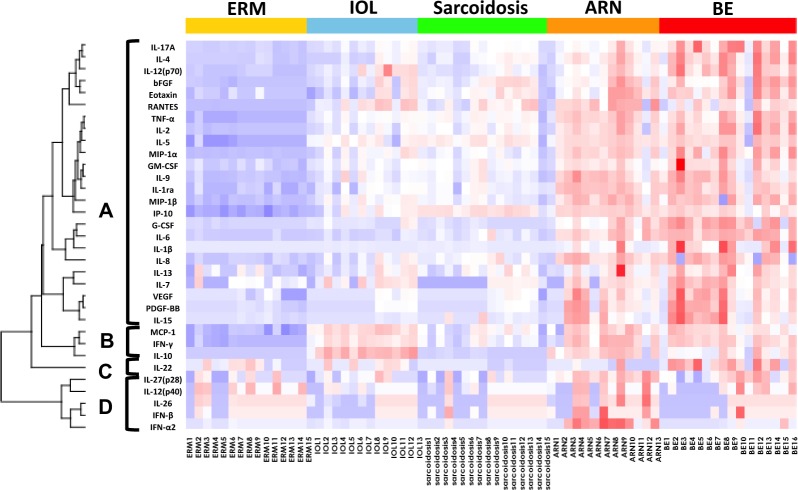
Heat map of inflammatory mediator concentrations in vitreous humor created using supervised hierarchical clustering with the x-axis fixed according to the disease components. The mediator is indicated by the label on the left. The mediators are separated into four main clusters (**A**–**D**) shown on the left. Group A includes mediators that were particularly elevated in both ARN and BE, group B includes those that were particularly elevated in IOL, ARN, and BE, group C includes those that are particularly elevated in BE, and group D includes those that are particularly elevated in ARN. ARN, acute retinal necrosis; BE, bacterial endophthalmitis; bFGF, basic fibroblast growth factor; ERM, idiopathic epiretinal membrane; G-CSF, granulocyte-colony stimulating factor; GM-CSF, granulocyte macrophage-colony stimulating factor; IFN, interferon; IL, interleukin; IOL, intraocular lymphoma; IP-10, interferon gamma-inducible protein-10; MCP, monocyte chemoattractant protein; MIP, macrophage inflammatory protein; PDGF, platelet-derived growth factor; RANTES, regulated on activation, normal T cell expressed and secreted; TNF, tumor necrosis factor; VEGF, vascular endothelial growth factor.

Table 3 summarizes the concentrations of the 33 inflammatory mediators identified in all 71 patients in this study. Figure 1 shows a heat map of these inflammatory mediators analysed by supervised hierarchical clustering of the 5 disease entities, in which group A constitutes mediators particularly elevated in both ARN and BE, group B those elevated in IOL, ARN and BE, group C those elevated particularly in BE, and group D those elevated particularly in ARN. A heat map analysis using unsupervised hierarchical clustering of the patients according to the similarities in their inflammatory mediator profiles identified 6 main groups: clusters I and II, which included all patients with ERM (n = 15); cluster III, which included most cases of ARN (8 of 13); cluster IV, which included most cases of BE (13 of 16); cluster V, which included most cases of sarcoidosis (13 of 15); and cluster VI, which included all cases of IOL (all 13 samples from 12 patients; Supplementary Fig. S1).

**Table 3:** Immune mediator levels in vitreous samples.

	ERM			IOL			sarcoidosis			ARN			BE		
	Concentra-tion, pg/ml	Range, pg/ml*	No. detected (%)	Concentra-tion, pg/ml	Range, pg/ml*	No. detected (%)	Concentra-tion, pg/ml	Range, pg/ml*	No. detected (%)	Concentration, pg/ml	Range, pg/ml*	No. detected (%)	Concentra-tion, pg/ml	Range, pg/ml*	No. detected (%)
IL-1β	0.02 ± 0.03	0 to 0.09	7 (46.7)	0.08 ± 0.09	0 to 0.27	8 (61.5)	0.12 ± 0.14	0 to 0.36	8 (53.3)	35.84 ± 119.45	0 to 449.54	11 (84.6)	930.98 ± 2532.17	0 to 10222.97	13 (81.3)
IL-1ra	5.47 ± 7.47	0 to 20.44	6(40)	103.73 ± 107.18	0 to 346.04	10(76.9)	105.29 ± 75.37	0 to 291.41	14(93.3)	9058.68 ± 9836.88	18.7 to 39538.95	13(100)	12730.9 ± 16147.67	46.13 to 54752.61	16(100)
IL-2	0.21 ± 0.27	0 to 0.85	7(46.7)	2.82 ± 1.62	0.21 to 5.87	13(100)	3.86 ± 2.06	0 to 7.13	14(93.3)	14.18 ± 11.04	0 to 45.38	12(92.3)	35.92 ± 42.51	0 to 162.07	15(93.8)
IL-4	0.08 ± 0.14	0 to 0.46	5(33.3)	0.56 ± 0.66	0 to 1.78	7(53.8)	0.84 ± 0.64	0 to 1.99	12(80)	2.1 ± 1.63	0 to 6.73	12(92.3)	5.22 ± 4.42	0.26 to 15.49	16(100)
IL-5	0.59 ± 1.75	0 to 6.96	3(20)	23.54 ± 15.17	0 to 55	12(92.3)	30.66 ± 18.03	0 to 62.72	14(93.3)	51.17 ± 21.98	16.1 to 82.33	13(100)	65.18 ± 36.77	0 to 137.34	15(93.8)
IL-6	0.53 ± 0.7	0 to 2.24	8(53.3)	2.28 ± 2.12	0 to 6.42	12(92.3)	18.69 ± 47.38	0 to 194.53	14(93.3)	484.88 ± 617.2	1.48 to 2016.6	13(100)	1611.79 ± 1193.73	13.39 to 4631.37	16(100)
IL-7	10.39 ± 8.61	0 to 23.71	11(73.3)	24.03 ± 21.11	0 to 62.31	11(84.6)	12.27 ± 13.96	0 to 30.99	7(46.7)	26.19 ± 20.6	0 to 66.95	10(76.9)	51.84 ± 31.17	3.21 to 127.61	16(100)
IL-8	8.64 ± 5.61	1.95 to 20.28	15(100)	127.75 ± 186.8	2.75 to 616.59	13(100)	161.86 ± 326.57	9.74 to 1351.11	15(100)	3770.83 ± 5507.18	5.49 to19919.73	13(100)	5997.56 ± 7794.15	0 to 28017.91	13(81.3)
IL-9	5.96 ± 2.71	2.76 to 9.8	15(100)	10.1 ± 5.16	3.81 to 18.83	13(100)	10.01 ± 3.76	4.35 to 15.8	15(100)	22.01 ± 10.23	3.54 to 40.72	13(100)	22.59 ± 9.34	9.43 to 39.19	16(100)
IL-10	0.56 ± 0.6	0 to 1.32	7(46.7)	780.3 ± 684.26	13.33 to 1849.0	13(100)	1.26 ± 1.3	0 to 3.45	8(53.3)	194.88 ± 135.22	0 to 452.3	12(92.3)	100.19 ± 197.71	0 to 720.26	15(93.8)
IL-12(p40)	9.16 ± 13.71	0 to 49.43	7(46.7)	2.15 ± 3.21	0 to 8.53	6(46.2)	2.06 ± 5.24	0 to 20.41	4(26.7)	10.22 ± 13.48	0 to 34.03	6(46.2)	3.7 ± 6.56	0 to 25.83	6(37.5)
IL-12(p70)	1.93 ± 0.91	0.93 to 3.19	15(100)	5.28 ± 5.34	1.26 to 20.88	13(100)	2.96 ± 1.77	1.17 to 6.55	15(100)	4.82 ± 3.34	1.26 to 11.8	13(100)	8.26 ± 5.22	2.19 to 23.28	16(100)
IL-13	1.16 ± 0.99	0.05 to 3.2	15(100)	1.69 ± 1.18	0.34 to 4.15	13(100)	1.72 ± 1.01	0.45 to 4.15	15(100)	5.01 ± 10.08	0.76 to 39.72	13(100)	3.85 ± 2.51	1.13 to 11.13	16(100)
IL-15	16.32 ± 15.76	0 to 39.93	8(53.3)	14.77 ± 18.83	0 to 42.7	5(38.5)	17.64 ± 18.47	0 to 48.05	9(60)	27.85 ± 25.84	0 to 85.74	11(84.6)	51.99 ± 48.25	0 to 186.34	15(93.8)
IL-17A	1.79 ± 0.58	0 to 66.44	15(100)	5.38 ± 3.18	2.08 to 10.86	13(100)	5.81 ± 2.82	2.19 to 10.08	15(100)	13.6 ± 18.26	1.98 to 74.92	13(100)	104.33 ± 115.61	5.6 to 386.7	16(100)
IL-22	4.54 ± 7.73	0 to 29.46	8(53.3)	4.77 ± 5.6	0 to 14.48	6(46.2)	0.05 ± 0.18	0 to 0.74	1(6.7)	7.75 ± 26.27	0 to 98.72	2(15.4)	75.33 ± 122.21	0 to 388.63	12(75)
IL-26	6.91 ± 13.55	0 to 48.92	4(26.7)	0.48 ± 0.85	0 to 2.84	4(30.8)	0.99 ± 3.71	0 to 14.87	1(6.7)	11.27 ± 14.95	0 to 39.79	6(46.2)	0 ± 0	0 to 0	0(0)
IL-27(p28)	15.86 ± 26.6	0 to 98.32	6(40)	9.63 ± 10.75	0 to 35.43	9(69.2)	10.79 ± 24.98	0 to 101.16	6(40)	67.51 ± 62.77	4.7 to 169.92	13(100)	25.27 ± 27.97	0 to 95.15	13(81.3)
IFN-α2	0.38 ± 0.98	0 to 3.82	3(20)	0.26 ± 0.36	0 to 1.15	6(46.2)	0.34 ± 1.29	0 to 5.16	1(6.67)	271.51 ± 476.3	0 to 1817.61	10(76.9)	1.13 ± 3.71	0 to 15.3	3(18.8)
IFN-β	1.41 ± 3.25	0 to 12.71	4(26.7)	0.08 ± 0.28	0 to 1.04	1(7.7)	1.09 ± 3.17	0 to 12.71	3(20)	7.81 ± 16.81	0 to 62.63	6(46.2)	1.01 ± 3.22	0 to 13.18	2(12.5)
IFN-γ	1.68 ± 2.75	0 to 10.77	7(46.7)	67.15 ± 52.92	12.47 to 162.17	13(100)	18.35 ± 24.32	0 to 91.6	14(93.3)	138.04 ± 166.27	2.49 to 552.85	13(100)	100.03 ± 90.08	3.33 to 279.31	16(100)
TNF-α	0.65 ± 1.52	0 to 5.91	7(46.7)	32.07 ± 19.52	12.27 to 81.87	13(100)	52.73 ± 36.75	0 to 122.07	14(93.3)	479.22 ± 475.87	8.65 to 1826.87	13(100)	1339.35 ± 2890.22	7.57 to 11695.12	16(100)
Eotaxin	1.31 ± 1.48	0 to 4.06	8(53.3)	3.3 ± 2.25	0 to 6.78	12(92.3)	6.12 ± 3.29	0 to 11.75	14(93.3)	12.69 ± 8.91	1.31 to 28.5	13(100)	20.25 ± 14.47	0 to 54.02	15(93.8)
MCP-1	154.37 ± 69.01	48.63 to 301.56	15(100)	1578.32 ± 457.03	854.83 to 2290.1	13(100)	620.06 ± 354.66	95.25 to 1319.53	15(100)	1574.5 ± 941.15	152.66 to 3068.65	13(100)	1446.65 ± 960	429.3 to 3860.85	16(100)
MIP-1α	0.3 ± 0.19	0.06 to 0.9	15(100)	12.82 ± 13.44	0.64 to 49.58	13(100)	6.55 ± 4.14	0.4 to 16.42	15(100)	53.21 ± 36.98	1.65 to 130.54	13(100)	241.41 ± 302.89	3.49 to 884.17	16(100)
MIP-1β	2.67 ± 1.4	1.01 to 6.18	15(100)	40.65 ± 31.12	5.6 to 120.42	13(100)	23.58 ± 16.49	2.16 to 63.63	15(100)	322.37 ± 258.5	9.42 to 776.54	13(100)	398.41 ± 340.02	0 to 1124.75	15(93.8)
RANTES	1.33 ± 1.42	0 to 3.06	7(46.7)	61.82 ± 49.68	4.08 to 144.49	13(100)	42.86 ± 55.12	0 to 215.97	14(93.3)	223.22 ± 180.06	0 to 616.08	12(92.3)	66.8 ± 89.54	0 to 361.84	15(93.8)
IP-10	206.61 ± 116.37	31.1 to 498.88	15(100)	13937.25 ± 13705.39	455.13 to 42474.68	13(100)	43792.28 ± 20600.5	1819.99 to 84799.71	15(100)	73053.02 ± 35931.77	5424.08 to 157774.68	13(100)	51506.76 ± 36371.15	3374.8 to 135519.56	16(100)
G-CSF	2.77 ± 3.28	0 to 9.11	7(46.7)	28.12 ± 32.13	0 to 100.64	10(76.9)	8.19 ± 10.97	0 to 39.04	8(53.3)	1368.33 ± 2735.63	8.39 to 9503.64	13(100)	102021.9 ± 194698.37	0 to 730999.14	14(87.5)
GM-CSF	0.86 ± 0.8	0 to 2.56	11(73.3)	1.67 ± 1.27	0.37 to 3.73	13(100)	2.01 ± 1.12	0.37 to 4.06	15(100)	3.08 ± 2.09	0.28 to 8.81	13(100)	12.03 ± 29.4	1 to 125.26	16(100)
bFGF	2.72 ± 3.02	0 to 7.01	7(46.7)	10.83 ± 5.71	0 to 23.89	12(92.3)	13.86 ± 9.73	0 to 44.21	14(93.3)	25.7 ± 15.27	1.16 to 59.5	13(100)	46.22 ± 42.45	0 to 176.59	15(93.8)
PDGF-BB	11.25 ± 11.53	0 to 30.32	8(53.3)	20.62 ± 26.17	0 to 58.59	5(38.5)	20.25 ± 23.84	0 to 62.06	7(46.7)	53.98 ± 48.38	0 to 168.46	10(76.9)	96.91 ± 90.06	0 to 314.82	15(93.8)
VEGF	8.8 ± 17.64	0 to 66.44	5(33.3)	23.66 ± 32.64	0 to 80.06	5(38.5)	28.55 ± 40.63	0 to 124.06	6(40)	230.87 ± 382.39	0 to 1140.5	10(76.9)	935.46 ± 2159.04	0 to 8999.37	15(93.8)

^*^The concentration of zero were assigned when the sample values were below the threshold and undetectable.

**Figure 2:**
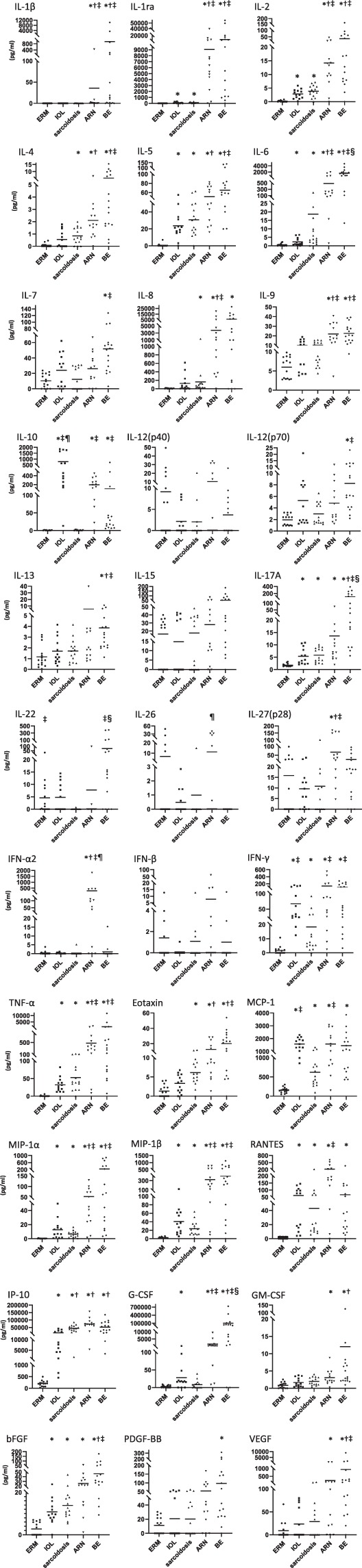
Scatter plots showing levels of immune mediators in the vitreous humor of eyes with ERM (controls without inflammation) and those with IOL, sarcoidosis, ARN, and BE. The concentrations of inflammatory mediators were compared between ERM and the other 4 diseases by stepwise comparisons using the Mann-Whitney *U* test followed by Bonferroni correction to compensate for multiple comparisons. A p-value < 0.005 was considered significant. The p-values are shown in the upper portion of each graph. *Significantly elevated when compared with ERM; ^†^significantly elevated when compared with IOL; ^‡^significantly elevated when compared with sarcoidosis; ^§^significantly elevated when compared with ARN; ^¶^significantly elevated when compared with BE. The horizontal line in the scatter plots represents the mean values. ARN, acute retinal necrosis; BE, bacterial endophthalmitis; ERM, idiopathic epiretinal membrane; IOL, intraocular lymphoma.

A multi-group statistical analysis using the Kruskal-Wallis test (Supplementary Table S2) revealed significant differences in the levels of 31 of the 33 inflammatory mediators (the exceptions being IL-12p40 and interferon [IFN]-β) between the 5 disease entities (p < 0.05). Scatter plots for all the mediators are shown in Fig. 2 and the pair-wise statistical data in Tables 4 and 5. Fifteen of the 33 mediators were significantly elevated in IOL, 16 in sarcoidosis, 24 in ARN, and 26 in BE when compared with those in ERM (p < 0.005; Table 4). There was no significant difference in the levels of IL-12p40, IL-15, IL-26, or IFN-β between any of the 4 disease groups with uveitis when compared with the ERM group. Pairwise comparisons using the Mann-Whitney *U* test with Bonferroni correction revealed that IFN-α2 in ARN and IL-6, IL-17A, and granulocyte-colony stimulating factor (G-CSF) in BE increased in an aetiology-specific manner (p < 0.005; Table 5). The IFN-α2 levels in ARN (271.5 ± 476.3 pg/ml, 77% detection) were respectively about 710-fold, 1000-fold, 800-fold, and 240-fold higher than those in the ERM (0.38 ± 0.98 pg/ml, 20%), IOL (0.26 ± 0.36 pg/ml, 46%), sarcoidosis (0.34 ± 1.29 pg/ml, 7%), and BE (1.13 ± 3.71 pg/ml, 19%) groups (Table 3). IL-6 and IL-17A levels in BE (1611.8 ± 1193.7 pg/ml and 104.3 ± 115.6 pg/ml, respectively, 100% detection of both) were respectively about 3000-fold and 58-fold, 710-fold and 19-fold, 86-fold and 18-fold, and 3.3-fold and 7.7-fold higher than those in ERM (0.53 ± 0.7 pg/ml and 1.79 ± 0.58 pg/ml, 53% and 100%), IOL (2.28 ± 2.12 pg/ml and 5.38 ± 3.18 pg/ml, 92% and 100%), sarcoidosis (18.7 ± 47.4 pg/ml and 5.81 ± 2.82 pg/ml, 93% and 100%) and ARN (484.9 ± 617.2 pg/ml and 13.6 ± 18.3 pg/ml, 100% in both). The G-CSF levels in BE (102.0 ± 194.7 ng/ml, 88% detection) were respectively about 37,000-fold, 3600-fold, 12,000, and 75-fold higher than those in ERM (2.77 ± 3.28 pg/ml, 47%), IOL (28.1 ± 32.1 pg/ml, 77%), sarcoidosis (8.19 ± 11.0 pg/ml, 53%), and ARN (1.37 ± 2.74 ng/ml, 100%), indicating that levels of G-CSF were more significantly increased than those of IL-6 and IL-17A in BE.

**Table 4:** Statistical data for immune mediators in the vitreous humor of patients with ERM and the other 4 uveitic diseases.

	IOL	sarcoidosis	ARN	BE
Cytokines	vs ERM			
IL-1β	0.0597	0.1298	**0.0002 ↑**	**0.0002 ↑**
IL-1ra	**0.0014 ↑**	**<0.0001 ↑**	**<0.0001 ↑**	**<0.0001 ↑**
IL-2	**<0.0001 ↑**	**<0.0001 ↑**	**<0.0001 ↑**	**<0.0001 ↑**
IL-4	0.0999	**0.004 ↑**	**<0.0001 ↑**	**<0.0001 ↑**
IL-5	**<0.0001 ↑**	**<0.0001 ↑**	**<0.0001 ↑**	**<0.0001 ↑**
IL-6	**0.0019 ↑**	**<0.0001 ↑**	**<0.0001 ↑**	**<0.0001 ↑**
IL-7	0.1429	0.9747	0.061	**<0.0001 ↑**
IL-8	0.0153	**<0.0001 ↑**	**<0.0001 ↑**	**0.0023 ↑**
IL-9	0.0355	0.0095	**<0.0001 ↑**	**<0.0001 ↑**
IL-10	**<0.0001 ↑**	0.1186	**<0.0001 ↑**	**<0.0001 ↑**
IL-12(p40)	0.4516	0.1444	0.8715	0.3442
IL-12(p70)	0.0556	0.082	0.0105	**<0.0001 ↑**
IL-13	0.1657	0.0993	0.0062	**<0.0001 ↑**
IL-15	0.9914	0.5418	0.1515	0.0103
IL-17A	**<0.0001 ↑**	**<0.0001 ↑**	**<0.0001 ↑**	**<0.0001 ↑**
IL-22	0.913	**0.0037 ↓**	0.0642	0.0069
IL-26	0.8466	0.1571	0.3023	0.0434
IL-27(p28)	0.5567	0.703	**0.0018 ↑**	0.1524
IFN-α2	0.2939	0.5977	**0.0002 ↑**	0.9481
IFN-β	0.1889	0.6513	0.2503	0.4191
IFN-γ	**<0.0001 ↑**	**<0.0001 ↑**	**<0.0001 ↑**	**<0.0001 ↑**
TNF-α	**<0.0001 ↑**	**<0.0001 ↑**	**<0.0001 ↑**	**<0.0001 ↑**
Eotaxin	0.0164	**<0.0001 ↑**	**<0.0001 ↑**	**<0.0001 ↑**
MCP-1	**<0.0001 ↑**	**<0.0001 ↑**	**<0.0001 ↑**	**<0.0001 ↑**
MIP-1α	** < 0.0001 ↑**	**<0.0001 ↑**	**<0.0001 ↑**	**<0.0001 ↑**
MIP-1β	**<0.0001 ↑**	**<0.0001 ↑**	**<0.0001 ↑**	**<0.0001 ↑**
RANTES	** < 0.0001 ↑**	**<0.0001 ↑**	**<0.0001 ↑**	**<0.0001 ↑**
IP-10	**<0.0001 ↑**	**<0.0001 ↑**	**<0.0001 ↑**	**<0.0001 ↑**
G-CSF	**0.0016 ↑**	0.2012	**<0.0001 ↑**	**0.0001 ↑**
GM-CSF	0.0993	0.0059	**0.0001 ↑**	**<0.0001 ↑**
bFGF	**<0.0001 ↑**	**<0.0001 ↑**	**<0.0001 ↑**	**<0.0001 ↑**
PDGF-BB	0.7944	0.5318	0.0053	**0.0001 ↑**
VEGF	0.4752	0.3672	**0.0026 ↑**	**<0.0001 ↑**

Represented are p-values generated by the Mann-Whitney U test followed by Bonferroni correction for multiple testing. Significant differences (p < 0.005) are in bold. ↑, significant increase compared with the controls (shown above), ↓, significant decrease compared with the controls.

**Table 5:** Statistical data for immune mediators in the vitreous humor of patients with IOL, sarcoidosis, ARN and BE.

Cytokines	IOL			sarcoidosis			ARN			BE		
	vs sarcoidosis	vs ARN	vs BE	vs IOL	vs ARN	vs BE	vs IOL	vs sarcoidosis	vs BE	vs IOL	vs sarcoidosis	vs ARN
IL-1β	0.5356	**0.0025 ↓**	**0.0012 ↓**	0.5356	**0.0031 ↓**	**0.0008 ↓**	**0.0025↑**	**0.0031↑**	0.0747	**0.0012↑**	**0.0008↑**	0.0747
IL-1ra	0.562	**<0.0001 ↓**	**<0.0001 ↓**	0.562	**<0.0001 ↓**	**<0.0001 ↓**	**<0.0001↑**	**<0.0001↑**	0.8123	**<0.0001↑**	**<0.0001↑**	0.8123
IL-2	0.1804	**0.0010 ↓**	**<0.0001 ↓**	0.1804	**0.0007 ↓**	**<0.0001 ↓**	**0.0010↑**	**0.0007↑**	0.4298	**<0.0001↑**	**<0.0001↑**	0.4298
IL-4	0.2503	**0.0023 ↓**	**<0.0001 ↓**	0.2503	0.0053	**<0.0001 ↓**	**0.0023↑**	0.0053	0.0368	**<0.0001↑**	**<0.0001↑**	0.0368
IL-5	0.42	**0.0029 ↓**	**0.0008 ↓**	0.42	0.0176	**0.0037 ↓**	**0.0029↑**	0.0176	0.4231	**0.0008↑**	**0.0037↑**	0.4231
*IL-6*	0.0293	**<0.0001 ↓**	**<0.0001 ↓**	0.0293	**0.0001 ↓**	**<0.0001 ↓**	**<0.0001↑**	**0.0001↑**	**0.0041↓**	***<0.0001↑***	***<0.0001↑***	***0.0041↑***
IL-7	0.0401	0.8901	0.0156	0.0401	0.0686	**0.0001 ↓**	0.8901	0.0686	0.0178	0.0156	**0.0001↑**	0.0178
IL-8	0.4672	**0.0002 ↓**	0.0087	0.4672	**0.0002 ↓**	0.0063	**0.0002↑**	**0.0002↑**	0.8459	0.0087	0.0063	0.8459
IL-9	0.8829	**0.0033 ↓**	**0.0003 ↓**	0.8829	**0.0004 ↓**	**<0.0001 ↓**	**0.0033↑**	**0.0004↑**	0.9828	**0.0003↑**	**<0.0001↑**	0.9828
IL-10	**<0.0001 ↑**	0.0102↑	**0.0003 ↑**	**<0.0001↓**	**<0.0001 ↓**	**<0.0001 ↓**	0.0102	**<0.0001↑**	0.0135	**0.0003↓**	**<0.0001↑**	0.0135
IL-12(p40)	0.3596	0.4196	0.9353	0.3596	0.1457	0.4382	0.4196	0.1457	0.3705	0.9353	0.4382	0.3705
IL-12(p70)	0.3204	0.5357	0.0347	0.3204	0.0817	**0.0004 ↓**	0.5357	0.0817	0.0389	0.0347	**0.0004↑**	0.0389
IL-13	0.8118	0.1566	**0.0033 ↓**	0.8118	0.2885	**0.0011 ↓**	0.1566	0.2885	0.0699	**0.0033↑**	**0.0011↑**	0.0699
IL-15	0.5842	0.0746	0.0078	0.5842	0.183	0.0165	0.0746	0.183	0.1837	0.0078	0.0165	0.1837
*IL-17A*	0.6747	0.0585	**<0.0001 ↓**	0.6747	0.0885	**<0.0001 ↓**	0.0585	0.0885	**0.0015 ↓**	***<0.0001↑***	***<0.0001↑***	***0.0015↑***
IL-22	0.01	0.1281	0.0082	0.01	0.3452	**<0.0001 ↓**	0.1281	0.3452	**0.0014 ↓**	0.0082↑	**<0.0001↑**	**0.0014↑**
IL-26	0.1528	0.2198	0.0301	0.1528	0.0131	0.4839	0.2198	0.0131	**0.0036 ↑**	0.0301	0.4839	**0.0036 ↓**
IL-27(p28)	0.2523	**0.0007 ↓**	0.1001	0.2523	**0.0001 ↓**	0.0173	**0.0007↑**	**0.0001↑**	0.0456	0.1001	0.0173	0.0456
*IFN-α2*	0.0286	**0.0023 ↓**	0.2134	0.0286	**<0.0001 ↓**	0.5328	***0.0023↑***	***<0.0001↑***	***0.0002 ↑***	0.2134	0.5328	**0.0002↓**
IFN-β	0.3833	0.0246	0.7154	0.3833	0.1118	0.7602	0.0246	0.1118	0.0562	0.7154	0.7602	0.0562
IFN-γ	**0.0008 ↑**	0.6139	0.5306	**0.0008 ↓**	**0.0026 ↓**	**0.0005 ↓**	0.6139	**0.0026↑**	0.9484	0.5306	**0.0005↑**	0.9484
TNF-α	0.116	**0.0008 ↓**	**<0.0001 ↓**	0.116	**0.0005 ↓**	**0.0008 ↓**	**0.0008↑**	**0.0005↑**	0.9828	**<0.0001↑**	**0.0008↑**	0.9828
Eotaxin	0.0278	**0.0006 ↓**	**0.0004 ↓**	0.0278	0.0464	**0.0049 ↓**	**0.0006↑**	0.0464	0.2495	**0.0004↑**	**0.0049 ↑**	0.2495
MCP-1	**<0.0001 ↑**	0.7241	0.268	**<0.0001 ↓**	**0.0031 ↓**	0.0055	0.7241	**0.0031↑**	0.6816	0.268	0.0055	0.6816
MIP-1α	0.4397	**0.0019 ↓**	**0.0012 ↓**	0.4397	**0.0004 ↓**	**<0.0001 ↓**	**0.0019↑**	**0.0004↑**	0.3291	**0.0012↑**	**<0.0001↑**	0.3291
MIP-1β	0.1329	**0.001 ↓**	**0.0025 ↓**	0.1329	**0.0002 ↓**	**0.0002 ↓**	**0.001↑**	**0.0002↑**	0.6816	**0.0025↑**	**0.0002↑**	0.6816
RANTES	0.2945	0.0072	0.7461	0.2945	**0.0045 ↓**	0.4766	0.0072↑	**0.0045↑**	0.0089↑	0.7461	0.4766	0.0089
IP-10	**0.0004 ↓**	**<0.0001 ↓**	**0.0015 ↓**	**0.0004↑**	0.0071	0.7405	**<0.0001↑**	0.0071	0.1697	**0.0015↑**	0.7405	0.1697
*G-CSF*	0.02	**0.0008 ↓**	**0.0002 ↓**	0.02	**<0.0001 ↓**	**<0.0001 ↓**	**0.0008↑**	**<0.0001↑**	**0.0021 ↓**	***0.0002↑***	***<0.0001↑***	***0.0021↑***
GM-CSF	0.3929	0.0699	**0.0046 ↓**	0.3929	0.1588	0.0148	0.0699	0.1588	0.1346	**0.0046 ↑**	0.0148	0.1346
bFGF	0.4063	0.0052	**0.0003 ↓**	0.4063	0.0129	**0.0013 ↓**	0.0052	0.0129	0.1991	**0.0003↑**	**0.0013↑**	0.1991
PDGF-BB	0.9509	0.0857	0.0065	0.9509	0.0633	0.0058	0.0857	0.0633	0.2724	0.0065	0.0058	0.2724
VEGF	0.9571	0.0181	**0.0002 ↓**	0.9571	0.0235	**0.0003 ↓**	0.0181	0.0235	0.1799	**0.0002↑**	**0.0003↑**	0.1799

Represented are the p-values from pairwise comparisons between 2 of the 4 uveitic diseases. The Mann-Whitney *U* test followed by Bonferroni correction for multiple testing between 5 diseases (including non-uveitis ERM, p < 0.005) or 3 diseases (intraocular lymphoma, acute retinal necrosis and bacterial endophthalmitis, p < 0.0166) were performed. Significant differences (p < 0.005) between the 5 diseases are in bold. The aetiology-specific immune mediators are highlighted in italic. The disease-specific immune mediators detected in IOL, ARN and BE (p < 0.0166) are highlighted in underline. ↑, Significant increase compared with the controls (shown above), ↓, significant decrease compared with the controls.

Furthermore, pairwise comparisons of IOL, ARN, and BE using Bonferroni correction showed that the vitreous levels of IL-10 in IOL, RANTES (regulated on activation, normal T cell expressed and secreted) in ARN, and IL-22 in BE were significantly higher than in the other 2 disease groups (p < 0.0166, Table 5, Fig. 2). The IL-10 level in IOL (780.3 ± 684.3 pg/ml, 100% detection) was respectively about 1400-fold, 620-fold, 4.0-fold, and 7.8-fold higher than that in ERM (0.56 ± 0.60 pg/ml, 47%), sarcoidosis (1.26 ± 1.30 pg/ml, 53%), ARN (194.9 ± 135.2 pg/ml, 92%), and BE (100.2 ± 197.7 pg/ml, 94%; Table 3). The level of RANTES in ARN (223.2 ± 180.1 pg/ml, 92%) was respectively about 170-fold, 3.6-fold, 5.2-fold, and 3.3-fold higher than that in ERM (1.33 ± 1.42 pg/ml, 47%), IOL (61.8 ± 49.7 pg/ml, 100%), sarcoidosis (42.9 ± 55.1 pg/ml, 93%), and BE (66.8 ± 89.5 pg/ml, 94%). The level of IL-22 in BE (75.3 ± 122.2 pg/ml, 75%) was respectively about 17-fold, 16-fold, 1500-fold, and 9.7-fold higher than that in ERM (4.54 ± 7.73 pg/ml, 53%), IOL (4.77 ± 5.60 pg/ml, 46%), sarcoidosis (0.05 ± 0.18 pg/ml, 7%), and ARN (7.75 ± 26.3 pg/ml, 15%).

Next, we examined the relationship between inflammatory mediator levels and severity of intraocular inflammation at the time of sample collection. The severity of intraocular inflammation at this time was determined by grading cells in the anterior chamber using a semi-quantitative scoring system (0 to 4+; described later in the Patients and Methods section). The 72 eyes were divided according to the cells in the anterior chamber into a 0–1 cell group (1+ or less) and a 2–4 cell group (2+ or more), and the concentrations of 33 mediators were compared between the two groups (Supplementary Fig. S2). Most of the mediators (27/33) were present in significantly higher concentrations in the 2–4 cell group than in the 0–1 cell group, whereas those of IL-10 and IL-26 were significantly higher in the 0–1 cell group. Moreover, the eyes were divided into the vitreous haze (VH) into a VH 0–1 group (1+ or less) and a VH 2–4 group (2+ or more), and the concentrations of 33 mediators were compared between the two groups (Supplementary Fig. S3). Majority of the mediators (20/33) had significantly higher concentrations in VH 2–4 group than in VH 0–1 group.

Furthermore, after finding large differences in inflammatory mediator levels between the group with infectious uveitis (ARN and BE) and the group with non-infectious uveitis (IOL and sarcoidosis), we performed supervised hierarchical clustering across the inflammatory mediator expression levels in each group separately to identify the disease-specific mediators in each group (Supplementary Figs S4 and S5). In the infectious group, there appeared to be higher expression levels of smaller clusters of cytokines (IL-27p28, IL-12p40, IL-26, IFN-α2, IFN-β, enclosed by the red line) in the eyes with ARN compared to those with BE (Supplementary Fig. S4). In the non-infectious group, there seemed to be a difference in the expression levels of other cytokines (MCP-1, IFN-γ, and IL-10) between the eyes with IOL and those with sarcoidosis (Supplementary Fig. S5).

## Discussion

In this study, we examined the vitreous concentrations of immune mediators in eyes with uveitis caused by IOL, sarcoidosis, ARN, or BE and in control eyes (with ERM, a disease that does not include uveitis). Our data suggest that the aetiology-specific inflammatory mediators that are elevated in the disease entities associated with uveitis included in this study are IL-10 in IOL, RANTES and IFN-α2 in ARN, and IL-6, IL-17A, IL-22, and G-CSF in BE. These mediators may be involved in the immunopathology of uveitis in different disease entities and contribute to the differences in its clinical characteristics.

At first, we demonstrated that the vitreous humor showed similar patterns of inflammatory mediators in eyes with uveitis depending on the aetiology (Fig. 1). The immune mediators in group A consisted mainly of tumor necrosis factor alpha (TNF-α) and a variety of inflammatory cytokines and chemokines produced by peripheral blood leukocytes when stimulated by TNF-ɑ, including IL-1 receptor antagonist, IL-1β, IL-6, IL-8, IL-17A, RANTES, macrophage inflammatory protein (MIP)-1α, MIP-1β, and interferon gamma-inducible protein (IP)-10. Group B included IL-10 and IFN-ɣ, which are mainly T-cell/natural killer (NK) cell-derived mediators that are produced in response to stimulation by various pathogens or antigens and are also elevated in the vitreous fluid of eyes with IOL. Group C consisted of IL-22, which is produced by dendritic cells, NK cells, and T helper 17 (T_H_17) cells in response to stimulation by bacterial pathogens and initiates the innate immune response to bacteria^15,16^. Group D included IFN-α2, IFN-β, and IL-26, which are mainly produced by virus-infected cells and play a role in the defence against viral infection^17^. Mediators involved in combatting bacteria (IL-22) and viruses (IFN-α2) were specifically elevated in BE and ARN. Levels of most of the immune mediators were high in BE and/or ARN. A few mediators, such as IL-10, were particularly elevated in IOL. None were selectively elevated in idiopathic ERM or sarcoidosis (Fig. 2).

The differences in levels of inflammatory mediators in the vitreous humor between ARN and BE are of interest because these are the most severe types of IU but have different clinical features. Compared with ARN, BE usually shows more severe intraocular inflammation, i.e., acute iridocyclitis with hypopyon, dense vitritis, and widespread necrotising retinitis, and progresses more rapidly. We found that IL-6, IL-17A, IL-22, and G-CSF levels were significantly higher in BE than in ARN, whereas the level of IFN-α2 was significantly higher in ARN than in BE (p < 0.005, Table 5, Fig. 2). Our present findings clearly reflect the role of these mediators in uveitis, particularly IU.

In the presence of bacterial infection, breaching of the epithelial barrier by bacteria stimulates dendritic cells, which in turn activate naive T-cells in the local draining lymph nodes^15^. IL-6 requires differentiation of naive T-cells to T_H_17 cells. Activated T_H_17 cells are recruited to the inflamed epithelium. Both T_H_17 cells and local dendritic cells secrete IL-22 to promote repair of the epithelium and also secrete antibacterial proteins. IL-17 secreted by T_H_17 cells induces epithelial cells to recruit neutrophils, which may also aid in removal of invading bacteria^15,16^. G-CSF stimulates the proliferation, differentiation, and function of neutrophils, thereby contributing to elimination of bacteria^18^. All mediators specifically elevated in the vitreous fluid of eyes with BE are essential for the host defence against bacterial pathogens. In contrast, IFN-α2, which was specifically elevated in ARN, polarises the immune system towards a specific type of response in order to combat viral infection^17^.

IOL showed a unique pattern of elevated inflammatory mediators in that only the IL-10 level was higher than in the other two disease entities. Previous studies have reported that IL-10, MIG (monokine induced by gamma interferon), B-cell-attracting chemokine-1, basic fibroblast growth factor (bFGF), IFN-γ, IL-6, and IL-8 are elevated in the vitreous humor of eyes with IOL^19,20^. However, in the present study, IL-10 was selectively increased in IOL whereas bFGF, IFN-γ, IL-6, and IL-8 were also elevated in sarcoidosis, ARN, and BE. In the same way, many cytokines (including IL-1 receptor antagonist, IL-6, IL-8, IFN-γ, and TNF-α) and chemokines (IP-10, MIP-1α, MIP-1β, and RANTES) have been reported to be present in higher concentrations in the vitreous humor of eyes with sarcoidosis than that in those with ERM^21,22^. However, we did not detect any inflammatory mediators that were elevated only in sarcoidosis. Immune mediators that had previously been reported to be elevated in the vitreous humor in IOL^19,20^ and sarcoidosis^21,22^ were present at markedly higher levels in ARN and BE than in IOL and sarcoidosis (Fig. 2). Therefore, most of the mediators thought to be elevated in IOL and sarcoidosis would not be useful biomarkers for uveitis with these aetiologies.

Our data support the suggestion that the IL-10/IL-6 ratio is a diagnostic marker of IOL^23,24^ because the IL-10 level was about 620-fold, 4.0-fold, and 7.8-fold higher and the IL-6 level was about 8.2-fold, 210-fold, and 710-fold lower in IOL than in sarcoidosis, ARN, and BE, respectively (Table 3). Furthermore, we found that the G-CSF level was about 49-fold and 3600-fold lower in IOL than in ARN and BE and that it was about 2.8-fold and 63-fold higher than the IL-6 level in these two diseases (Table 3). Therefore, the IL-10/G-CSF ratio may also be useful for diagnosis of IOL and in particular for distinguishing between IOL and BE.

IL-10 was found to be relatively selective for IOL. However, the average IL-10 level in IOL was only 4-fold and 8-fold higher than that in ARN and BE, respectively. Furthermore, the IL-10 level was higher than 200 pg/ml in about 70% of the patients with IOL, 40% of those with ARN, and 12% of those with BE (Fig. 2), and this overlap decreased the specificity of IL-10 as an IOL-selective diagnostic marker. In contrast, IFN-α2 levels were selectively increased in patients with ARN. The IFN-α2 level was 1000-fold higher in ARN than in IOL while the IL-6 level was 210-fold higher in ARN than in IOL. Only 31% of patients with ARN had an IFN-α2 level less than 10 pg/ml, which overlapped with all of the patients with IOL (Fig. 2). Therefore, combination of the IL-10 level with the levels of IFN-α2, IL-6, and G-CSF could also be useful for diagnosis of IOL.

Cytokine levels have previously been measured mainly in the aqueous humor^25–31^ and rarely in the vitreous humor^19–22,32–34^ of eyes with uveitis. The concentrations of cytokines in the vitreous humor are higher than those in the aqueous humor and suggested to be more reliable markers of vitreoretinal disease^23,35^. Cytokines or inflammatory mediators that have been reported to be present at higher levels in the aqueous/vitreous humor include IL-6, IFN-γ, and IL-10 in ARN^32^ and G-CSF, IFN-γ, IL-1α, IL-1β, IL-6, IL-8, IP-10, monocyte chemoattractant protein (MCP)-1, and TNF-α in BE^33,34^. However, those reports compared the elevated levels of cytokines or mediators between one type of uveitis and a control group with non-uveitic disease (such as ERM^19–22^, macular hole^19^, cataract^32^, retinal detachment^34^, or diabetic retinopathy^22,34^), cadaveric eyes^32,33^, eyes with toxoplasma chorioretinitis^32^, or a group of patients with uveitis of varying aetiology^20,32^. In some cases^33^, the data included results from both the aqueous humor and vitreous fluid. There have been no comparisons of mediator levels in the vitreous humor of eyes with uveitis associated with different aetiologies. Therefore, our data are useful for understanding the immunopathology selective to uveitis associated with specific disease entities.

This study has several limitations. First, the number of eyes in each group was relatively small. Second, there were between-group differences in the age and sex distributions. The patients in the ARN group were younger than those in the other disease groups and there was a predominance of female patients in the IOL and sarcoidosis groups, probably as a result of the epidemiology of these diseases. Third, no inclusion criteria were set regarding current medical therapy, severity of disease, or days between onset and collection of the vitreous samples, which may have affected the concentrations of inflammatory mediators obtained. However, despite these limitations, we believe that the present findings provide important information regarding aetiology-specific inflammatory mediators in the vitreous humor of eyes with uveitis. These findings will need to be validated before the potential utility of these cytokine profiles as disease biomarkers can be assessed. Our study included only small numbers of patients so is best considered a pilot study for future studies to build on. Further research in a larger sample could identify more reliable disease-specific immune mediators that could assist in the diagnosis of uveitis using artificial intelligence. Moreover, further studies of these inflammatory mediators in animal models of autoimmune uveitis and infectious uveitis will help to clarify the importance of immunopathogenesis in uveitis.

In conclusion, we have demonstrated that the aetiology-specific inflammatory mediators elevated in different types of uveitis include IFN-α2 in ARN and IL-6, IL-17A, and G-CSF in BE. IL-10 in IOL, RANTES in ARN, and IL-22 in BE may also be candidate aetiology-specific mediators. These mediators may be involved in the immunopathology of uveitis in different disease entities and contribute to differences in its clinical characteristics. Some of these mediators may be useful diagnostic biomarkers in patients with uveitis.

## Patients and Methods

The study protocol was approved by the internal ethics committees of The University of Tokyo Hospital and National Defense Medical College Hospital and performed in accordance with the principles of the Declaration of Helsinki. Written informed consent was obtained from all patients prior to their participation in the study.

### Patients

The study included 57 eyes of 56 patients (29 male, 27 female) who underwent pars plana vitrectomy for diagnosis and/or treatment of active uveitis and who were finally diagnosed to have IOL, sarcoidosis, ARN, or BE in the uveitis clinic at the University of Tokyo Hospital or National Defense Medical College Hospital between December 2008 and September 2018. Twelve patients (13 eyes) were diagnosed with IOL, 15 patients (15 eyes) with sarcoidosis, 13 patients (13 eyes) with ARN, and 16 patients (16 eyes) with BE. The diagnosis of IOL^7^, sarcoidosis^36^, or ARN^5^ was made according to the relevant diagnostic criteria usually used in Japan. The diagnosis of ARN was based on detection of human herpes virus DNA in ocular fluid by PCR^37^. All patients with BE had acute or late-onset endophthalmitis after cataract surgery or endophthalmitis after trabeculectomy. The diagnosis of BE was based on matching of ocular symptoms suggesting bacterial aetiology, blood culture results, detection of bacterial DNA in vitreous humor samples by broad-range PCR^14^, and a response to antibiotics. Eyes with inactive uveitis and those in which a diagnosis of uveitis was uncertain were excluded. One patient with IOL provided two vitreous samples from both eyes that were obtained on different surgical days. Vitreous samples from 15 eyes (15 patients) with idiopathic ERM undergoing posterior vitrectomy were included as a control group (Table 2). All patients underwent diagnostic ophthalmic examinations, including best-corrected visual acuity, slit-lamp biomicroscopy, and funduscopy. A standard three-port, 23-gauge or 25-gauge vitrectomy was performed, and the vitreous humor was collected aseptically without dilution. The vitreous samples were stored immediately in a pre-sterilised microcentrifuge tube at −80 °C until assayed.

### Patients’ clinical details at the time of collection of vitreous samples

The patients’ clinical details at the time of collection of the vitreous samples were retrospectively examined. The data on timing of the vitrectomy in relation to the onset of symptoms, severity of inflammation in the anterior chamber, vitreous opacity and retinitis presence or absence of hypopyon, causative infectious agents, lens status (phakic or pseudophakic), medications used before vitrectomy (topical application, subconjunctival injection, and systemic administration of steroids and systemic antibacterial/antiviral agents) were collected from the medical records (Supplementary Table S1). The severity of ocular inflammation was graded according to the criteria established by the Standardization of Uveitis Nomenclature (SUN) Working Group^38^. Briefly, cells in the anterior chamber (maximum 4 points) were graded using a semi-quantitative scoring system of 6 grades (0, 0.5+, 1+, 2+, 3+, and 4+). Vitreous opacity (maximum 4 points) was evaluated using a semi-quantitative scoring system of 6 grades (0, trace, 1+, 2+, 3+, and 4+) based on the clarity of the optic disc, retinal vessels, and nerve fibre layers on fundus examination as reported by Nussenblatt *et al*.^39^. Retinitis (maximum 4 points) was evaluated using a 5-point grading system (0, 1+, 2+, 3+, and 4+) based on the retinal area that was inflamed on fundus examination as follows: normal, grade 0; limited in a quadrant, grade 1; spreading into 2 quadrants, grade 2; spreading into 3 quadrants, grade 3; spreading into 4 quadrants, grade 4.

### Multiplex immunoassay

Multiplex proteomic analysis was used to measure the levels of inflammatory cytokines, chemokines, and growth factors in the vitreous samples. The concentrations of 33 human mediators (IL-1β, IL-1 receptor antagonist, IL-2, IL-4, IL-5, IL-6, IL-7, IL-8, IL-9, IL-10, IL-12p40, IL-12p70, IL-13, IL-15, IL-17A, IL-22, IL-26, IL-27p28, IFN-α2, IFN-β, IFN-γ, TNF-α, eotaxin, MCP-1, MIP-1α, MIP-1β, RANTES, IP-10, G-CSF, granulocyte-macrophage-CSF, bFGF, PDGF-BB, and VEGF; Table 3) were measured using a multiplex assay instrument (MAGPIX; Bio-Rad Laboratories, Hercules, CA, USA) according to the manufacturer’s instructions. There were no samples in this study where concentrations were above the upper range that could be analysed.

### Heat map cluster analysis

The heat map was generated using the gplots package in R version 3.5.3 (R Foundation for Statistical Computing, Vienna, Austria) based on cytokine expression. The color-coded heatmap of the concentrations of the 33 inflammatory mediators provided a good overview of their profiles in the 5 disease entities. First, the heat map analysis was performed using unsupervised hierarchical clustering (Supplementary Fig. S1). Supervised hierarchical clustering was then performed (Fig. 1), whereby the x-axis was divided according to disease entity to identify the clusters of elevated cytokines associated with uveitis in each disease. Furthermore, we performed supervised hierarchical clustering across cytokine expression levels in the infectious uveitis group (ARN and BE) and non-infectious uveitis group (IOL and sarcoidosis) separately to identify the disease-specific cytokines in each group (Supplementary Figs. S4 and S5).

### Statistical analysis

The data are shown as the mean ± standard deviation. Because the cytokine concentrations were not normal distributed, we used the non-parametric statistical analyses such as Mann-Whitney’s U-test and Kruskal Wallis test. The multi-group statistical analysis of the 5 disease entities was performed using the Kruskal-Wallis test, which resulted in a critical value for significance of p < 0.05 (Supplementary Table S2). The concentrations of mediators were compared using the Mann-Whitney *U* test based on the pattern of data distribution; a p-value < 0.05 was considered statistically significant. The Mann-Whitney *U* test was repeated for each cytokine to identify mediators that might be present in higher concentrations in one type of eye disease than in the other diseases. A Bonferroni correction was implemented to counteract any type I error attributable to the multiple comparisons between the 5 diseases, which resulted in a critical value for significance of p < 0.005 (Tables 4 and 5). A Bonferroni correction was implemented in the pairwise comparisons between IOL, ARN, and BE, which resulted in a critical value for significance of p < 0.0166 (Table 5). Scatter plots were created using GraphPad Prism for Windows (version 8; GraphPad Software Inc., La Jolla, CA, USA). When the concentrations in the raw data were below the detection limit, they were coded as 0 and included in the statistical analysis. All the statistical analyses were performed using GraphPad Prism for Windows.

## Supplementary information

Supplementary information.

Supplementary information2

## Data Availability

The data showing the concentrations of immune mediators in the vitreous humor will be uploaded to Springer Nature’s Research Data Support service.
