# Supplementary material for: Analysis of inflammatory mediators in the vitreous humor of eyes with pan-uveitis according to aetiological classification
Source: Sci Rep. 2020 Feb 17;10:2783. doi: 10.1038/s41598-020-59666-0 (PMC7026072; doi:10.1038/s41598-020-59666-0)
Supplement: Supplementary file 1 — Supplementary information. [file 41598_2020_59666_MOESM1_ESM.pdf]

Supplementary Online Content

Analysis of inflammatory mediators in the vitreous humor of eyes with pan-uveitis according to aetiological classification

Hisako Fukunaga, Toshikatsu Kaburaki, Shintaro Shirahama, Rie Tanaka, Hiroshi Murata, Tomohito Sato, Masaru Takeuchi, Hideto Tozawa,

Yoshihiro Urade, Mari Katsura, Mika Kobayashi, Youichiro Wada, Hirotsugu Soga, Hidetoshi Kawashima, Takahide Kohro, Makoto Aihara

Table of Contents

Supplementary Fig. S1. Heat map of inflammatory mediator concentrations in vitreous humor created using non-supervised hierarchical clustering

Supplementary Fig. S2. Comparisons of concentrations of immune mediators according to severity of intraocular inflammation in anterior chamber

Supplementary Fig. S3. Comparisons of concentrations of immune mediators according to severity of vitreous haze

Supplementary Fig. S4. Heat map of inflammatory mediator concentrations in the vitreous humor in eyes with infectious uveitis (acute retinal necrosis and bacterial endophthalmitis)

Supplementary Fig. S5. Heat map of inflammatory mediator concentrations in the vitreous humor in eyes with non-infectious uveitis (intraocular lymphoma and sarcoidosis)

Supplementary Fig. S6. Comparisons of concentrations of immune mediators according to culture-positive and culture-negative cases

Supplementary Table S1. Patients' clinical details at the time of sample collection

Supplementary Table S2. Statistical data of immune mediators in vitreous humor of patients with ERM and other 4 uveitis diseases

Supplementary Data S1. The raw data for patients' clinical details at the time of sample collection

Supplementary Fig. S1. Heat map of inflammatory mediator concentrations in vitreous humor created using non-supervised hierarchical clustering. The mediator concentrations are depicted as colours ranging from blue to white to red, indicating low, intermediate, and high concentration, respectively, relative to the mean value for that cytokine. The label on the left of the heat map indicates cytokine expression. The disease entity associated with each sample is indicated by the label on the bottom as well as by the coloured bar on the top. The study eyes are separated in 6 main clusters (I to VI) separated by blue lines.

Supplementary Fig. S1

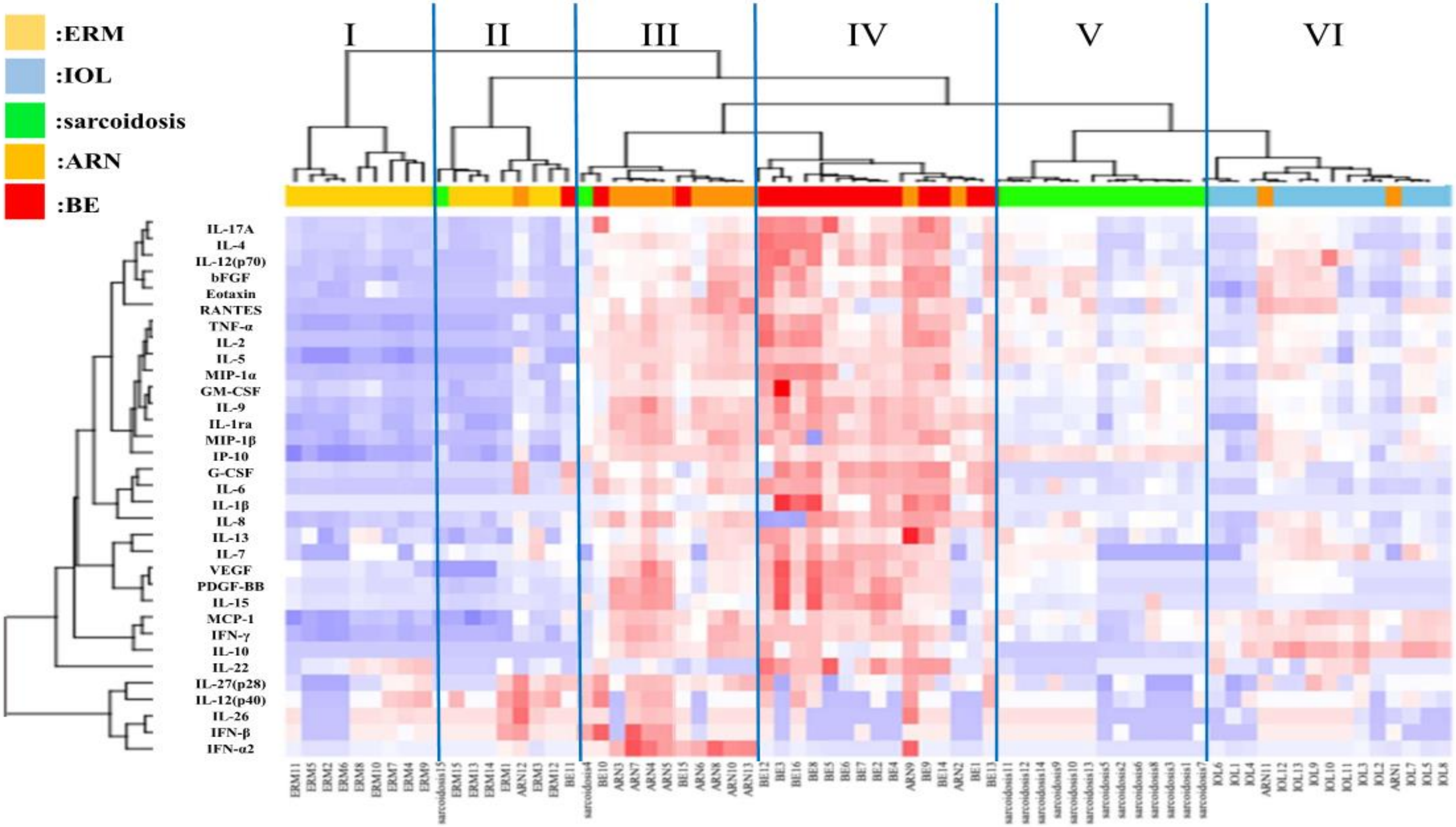

Supplementary Fig. S2. Comparisons of concentrations of immune mediators according to severity of intraocular inflammation in anterior chamber. The severity of intraocular inflammation in anterior chamber at the time of sample collection was determined using a semi-quantitative scoring system for cells in anterior chamber (0 to 4+) as described in the Patients and Methods. The eyes were divided according to the cells in the anterior chamber into a 0-1 cell group (1+ or less) and a 2-4 cell group (2+ or more), and the concentrations of 33 mediators were compared between the two groups. A p-value <0.05 was considered statistically significant. The p-values are shown in the upper portion of each graph. *p<0.05, **p<0.01, ***p<0.001, ****p<0.0001

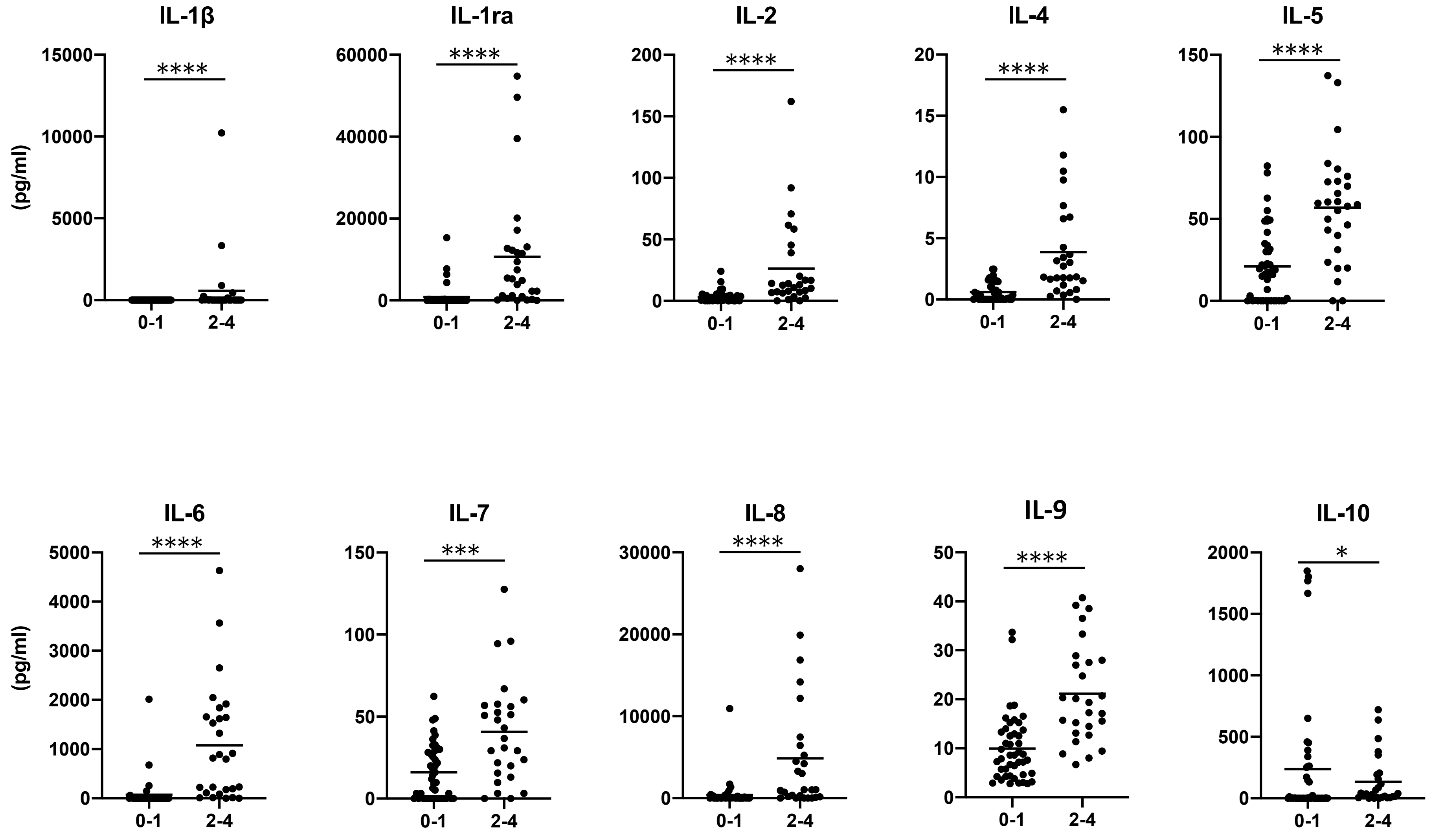

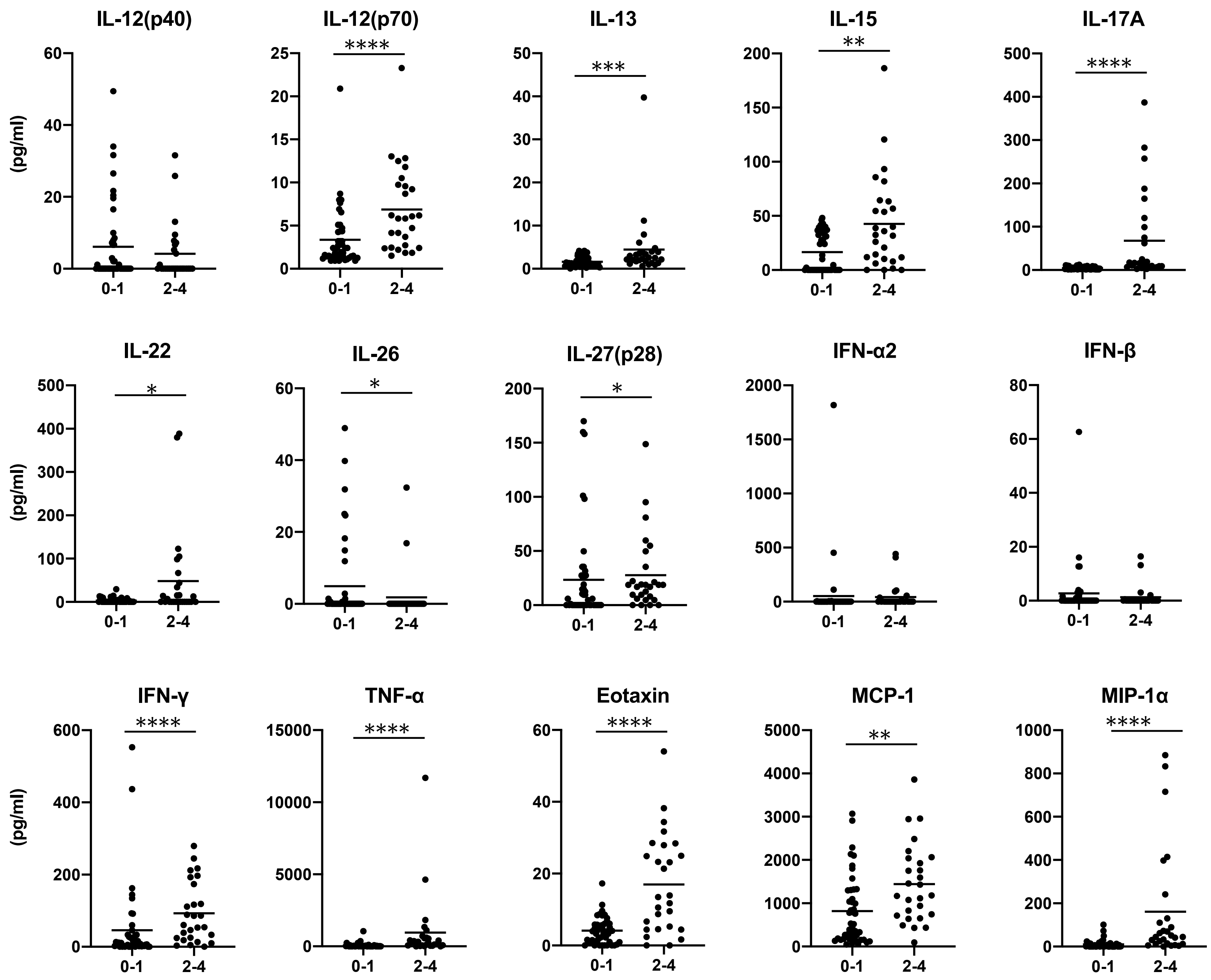
IL-12(p40)
IL-12(p70)
IL-13
IL-15
IL-17A
IL-22
IL-26
IL-27(p28)
IFN-α2
IFN-β
IFN-γ
TNF-α
Eotaxin
MCP-1
MIP-1α
(pg/ml)
0-1
2-4
****
***
**
*

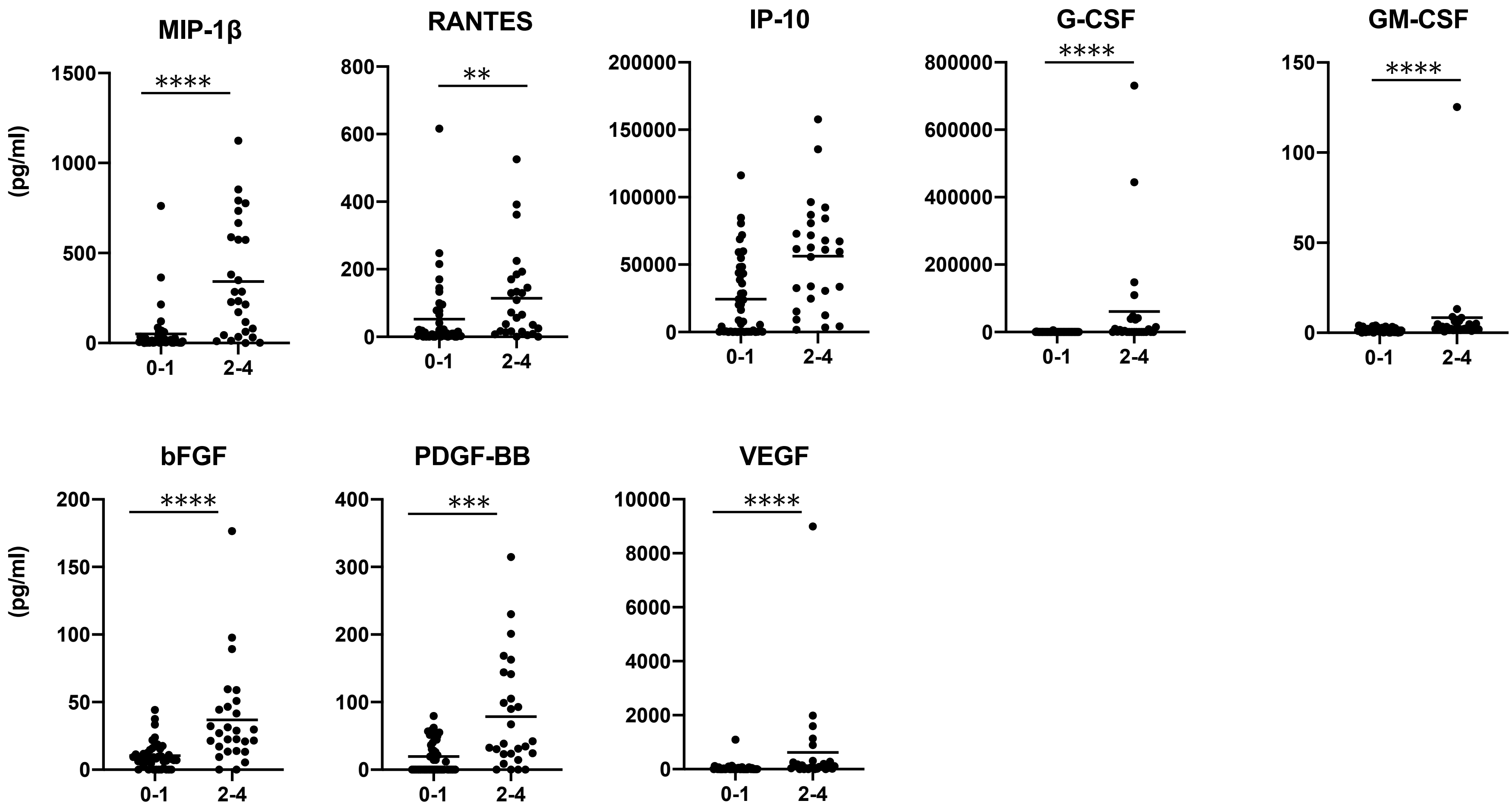

MIP-1β
RANTES
IP-10
G-CSF
GM-CSF
bFGF
PDGF-BB
VEGF
(pg/ml)
****
**
***
0-1
2-4

Supplementary Fig. S3. Comparisons of concentrations of immune mediators according to severity of vitreous haze. The severity of vitreous haze at the time of sample collection was determined using a semi-quantitative scoring system for vitreous haze (0 to 4+) as described in the Patients and Methods. The eyes were divided according to the vitreous haze (VH) into a VH 0-1 group (1+ or less) and a VH 2-4 group (2+ or more), and the concentrations of 33 mediators were compared between the two groups. A p-value <0.05 was considered statistically significant. The p-values are shown in the upper portion of each graph. *p<0.05, **p<0.01, ***p<0.001, ****p<0.0001

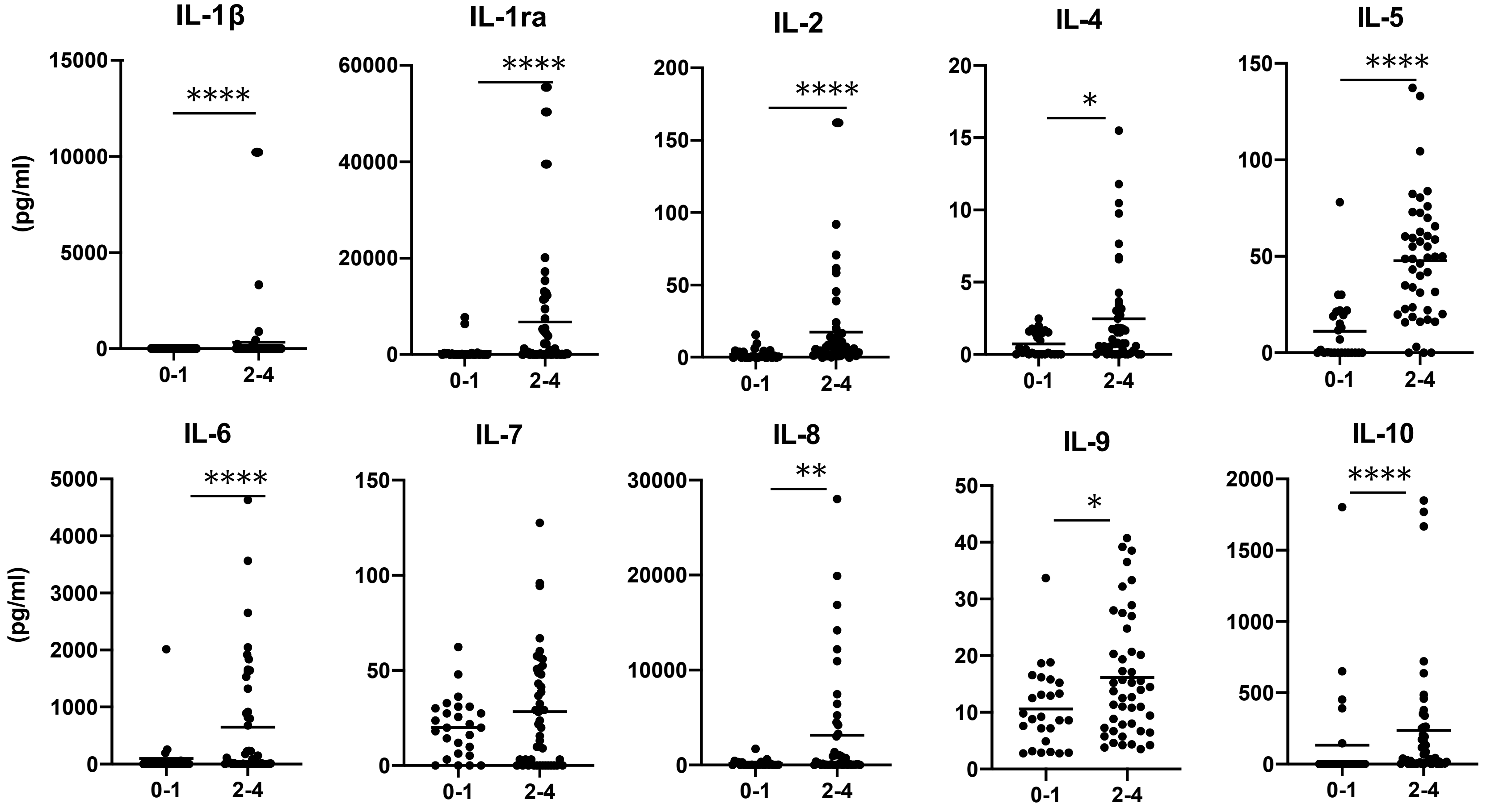

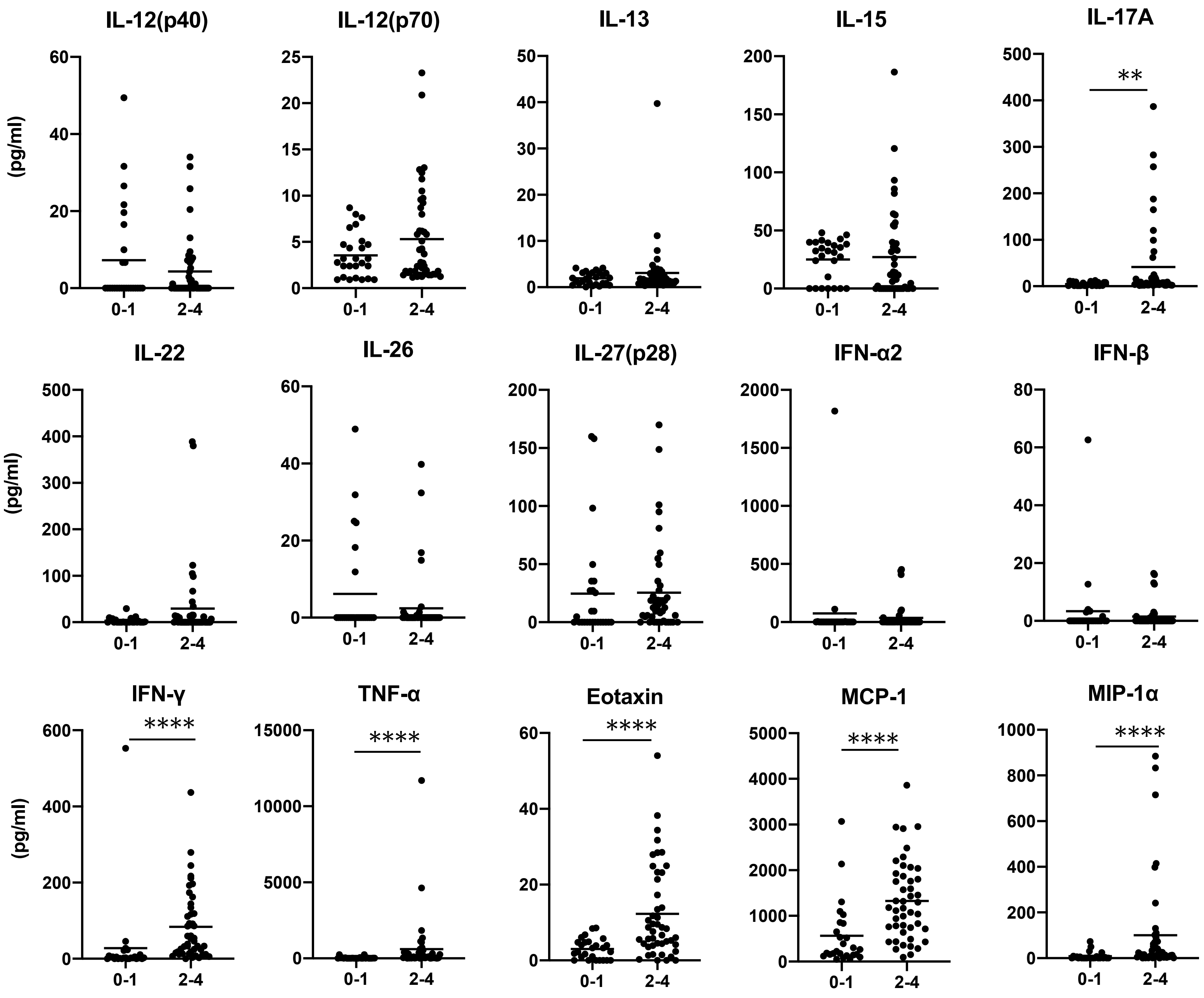

IL-12(p40)
IL-12(p70)
IL-13
IL-15
IL-17A
IL-22
IL-26
IL-27(p28)
IFN-α2
IFN-β
IFN-γ
TNF-α
Eotaxin
MCP-1
MIP-1α
(pg/ml)
0-1
2-4
**
****

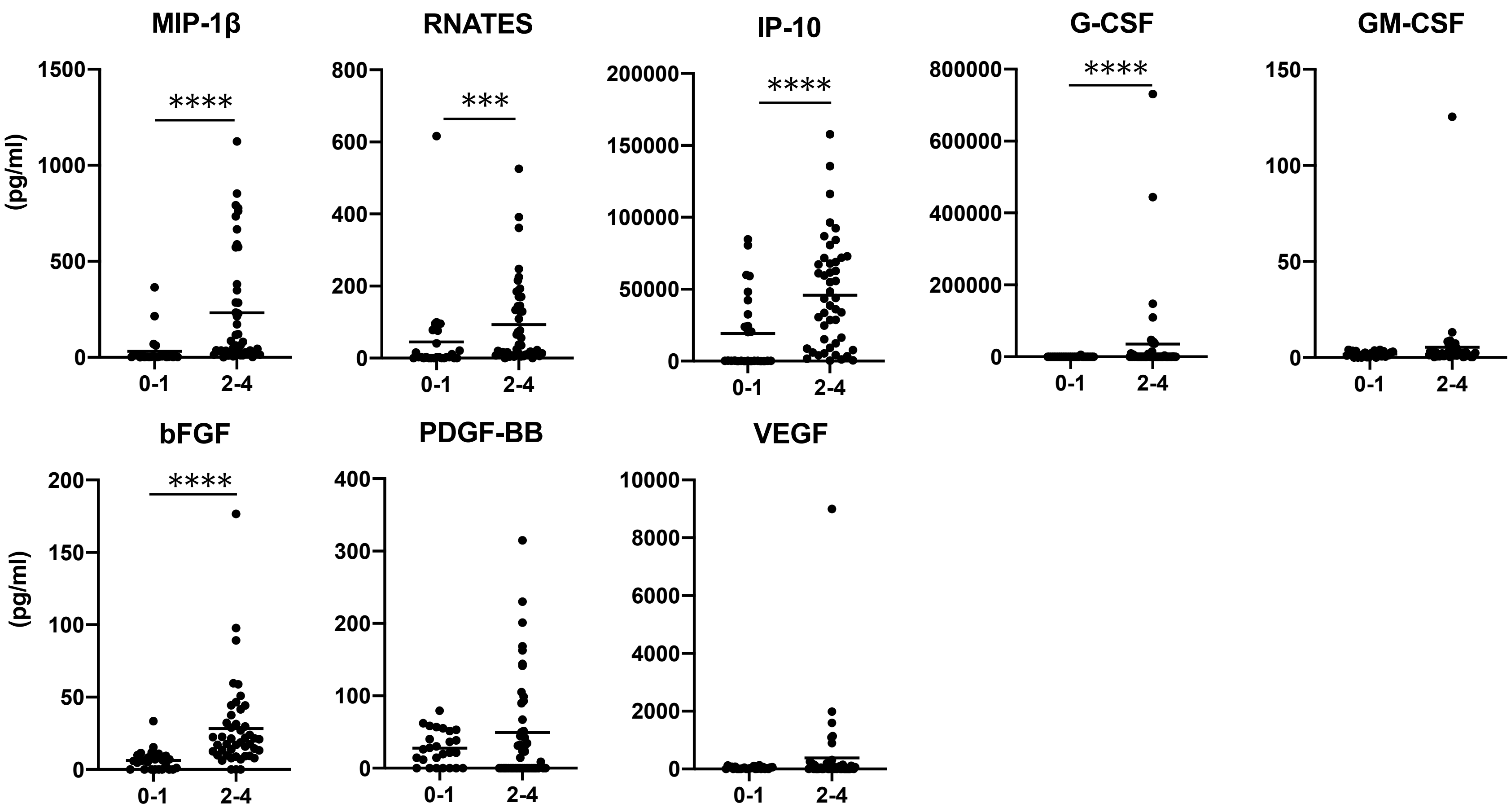

MIP-1β
RNATES
IP-10
G-CSF
GM-CSF
bFGF
PDGF-BB
VEGF
(pg/ml)
0-1
2-4
****
***

Supplementary Fig. S4. Heat map of inflammatory mediator concentrations in the vitreous humor in eyes with infectious uveitis (acute retinal necrosis and bacterial endophthalmitis) created using supervised hierarchical clustering with the x-axis fixed according to the disease components. The mediator is indicated by the label on the left. The cluster of cytokines with relatively higher expression in ARN are enclosed by the red line. ARN, acute retinal necrosis; BE, bacterial endophthalmitis

Supplementary Fig. S4

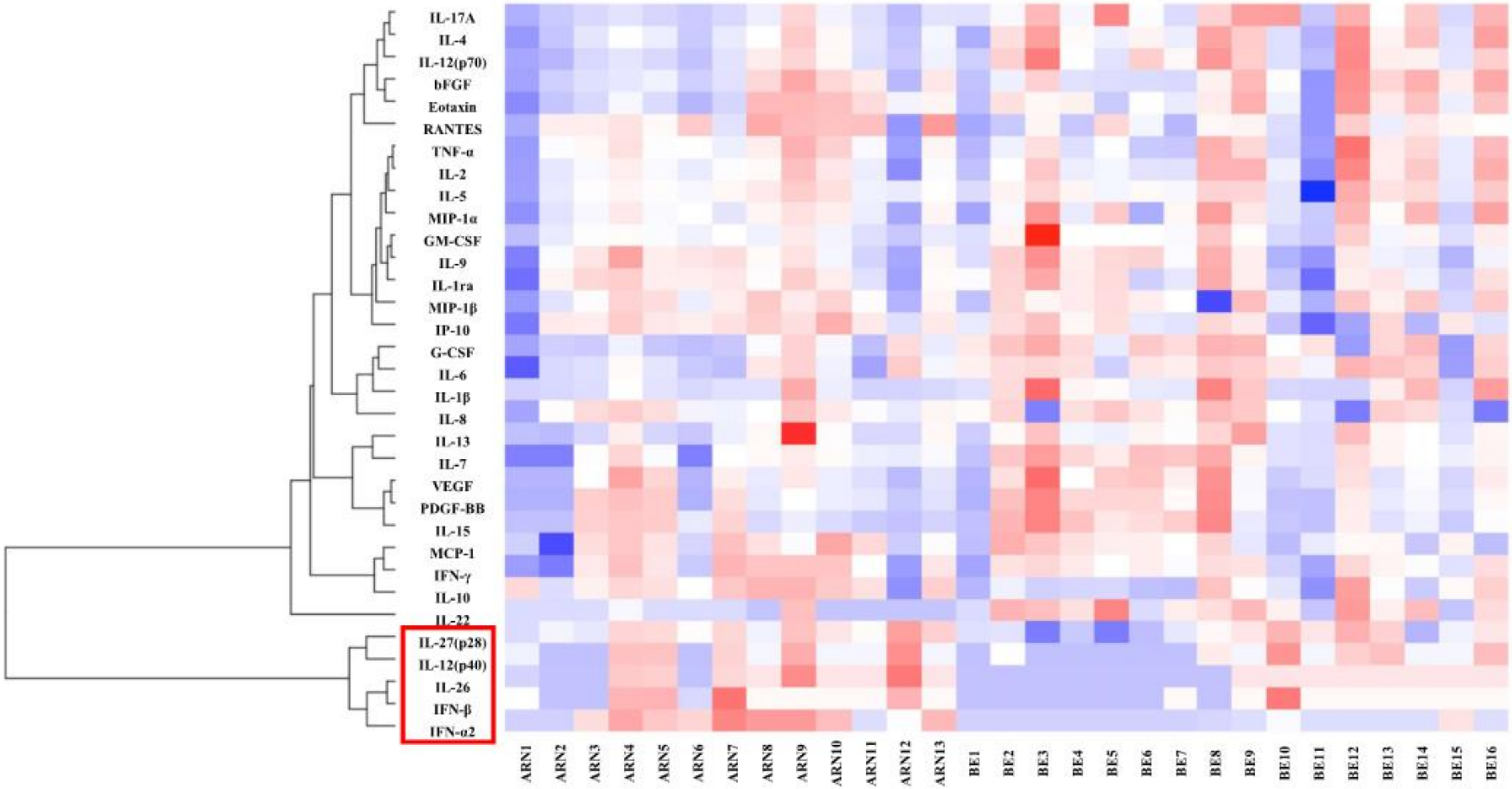

Supplementary Figure S5. Heat map of inflammatory mediator concentrations in the vitreous humor in eyes with non-infectious uveitis (intraocular lymphoma and sarcoidosis) created using supervised hierarchical clustering with the x-axis fixed according to the disease components. The mediator is indicated by the label on the left. The cluster of cytokines with relatively higher expression in IOL are enclosed by the red line. IOL, intraocular lymphoma

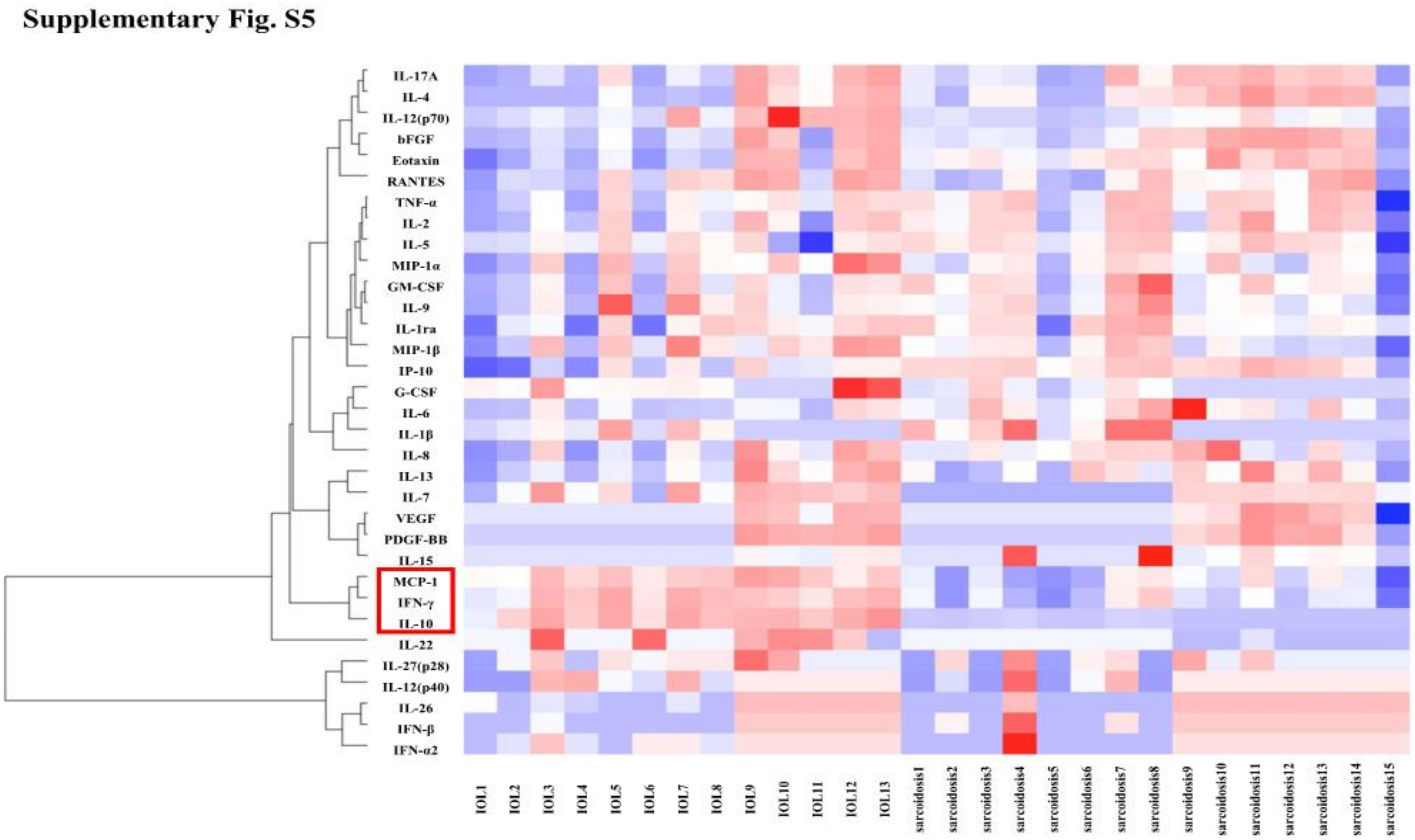

Supplementary Figure S6. Comparisons of concentrations of immune mediators according to culture-positive and culture-negative cases. The concentrations of 33 mediators were compared between **culture-positive and culture-negative cases in patients with bacterial endophthalmitis.** Open circles indicate cytokine concentrations in a case of bacterial endophthalmitis caused by Gram-negative bacteria. A p-value <0.05 was considered statistically significant.

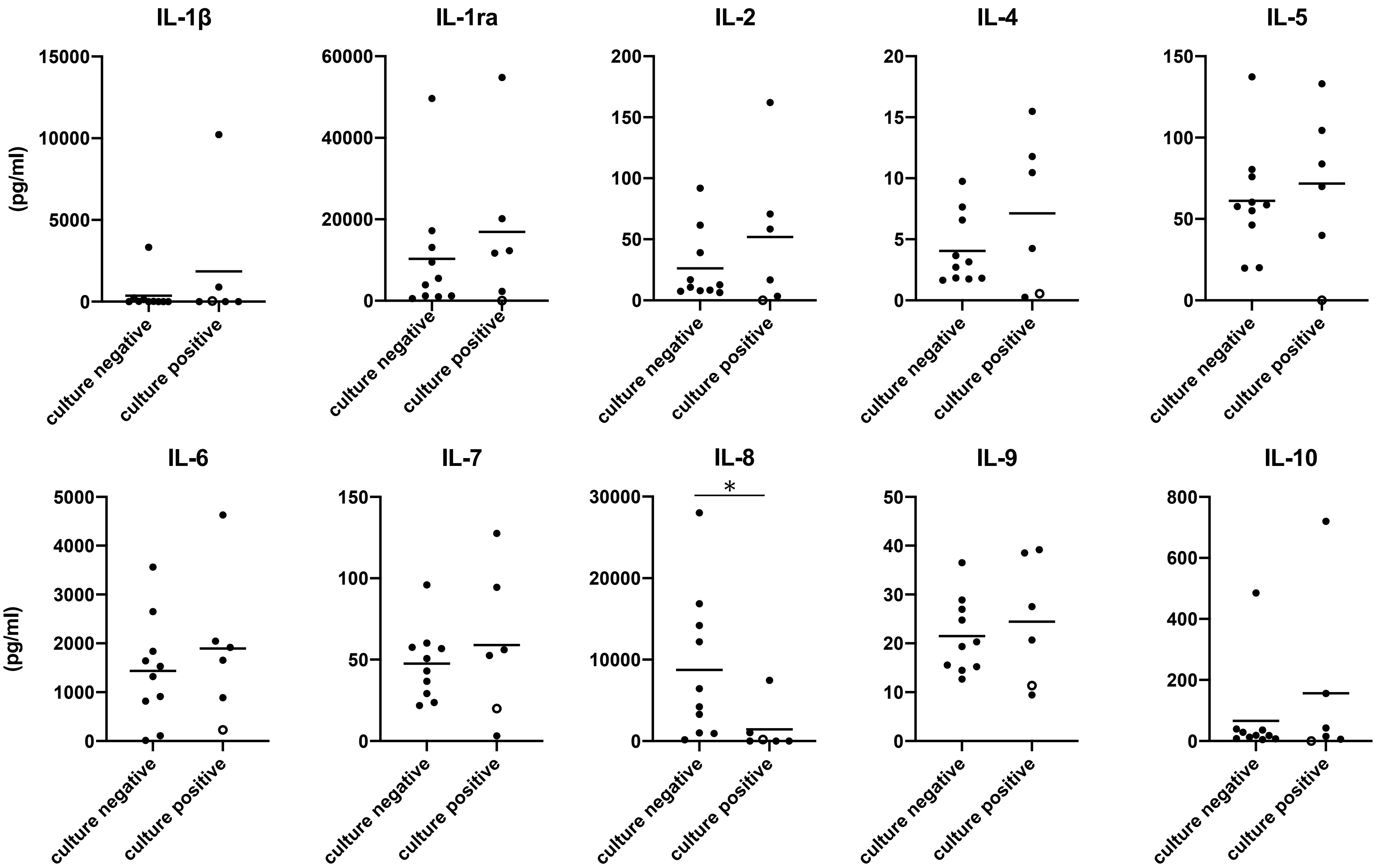

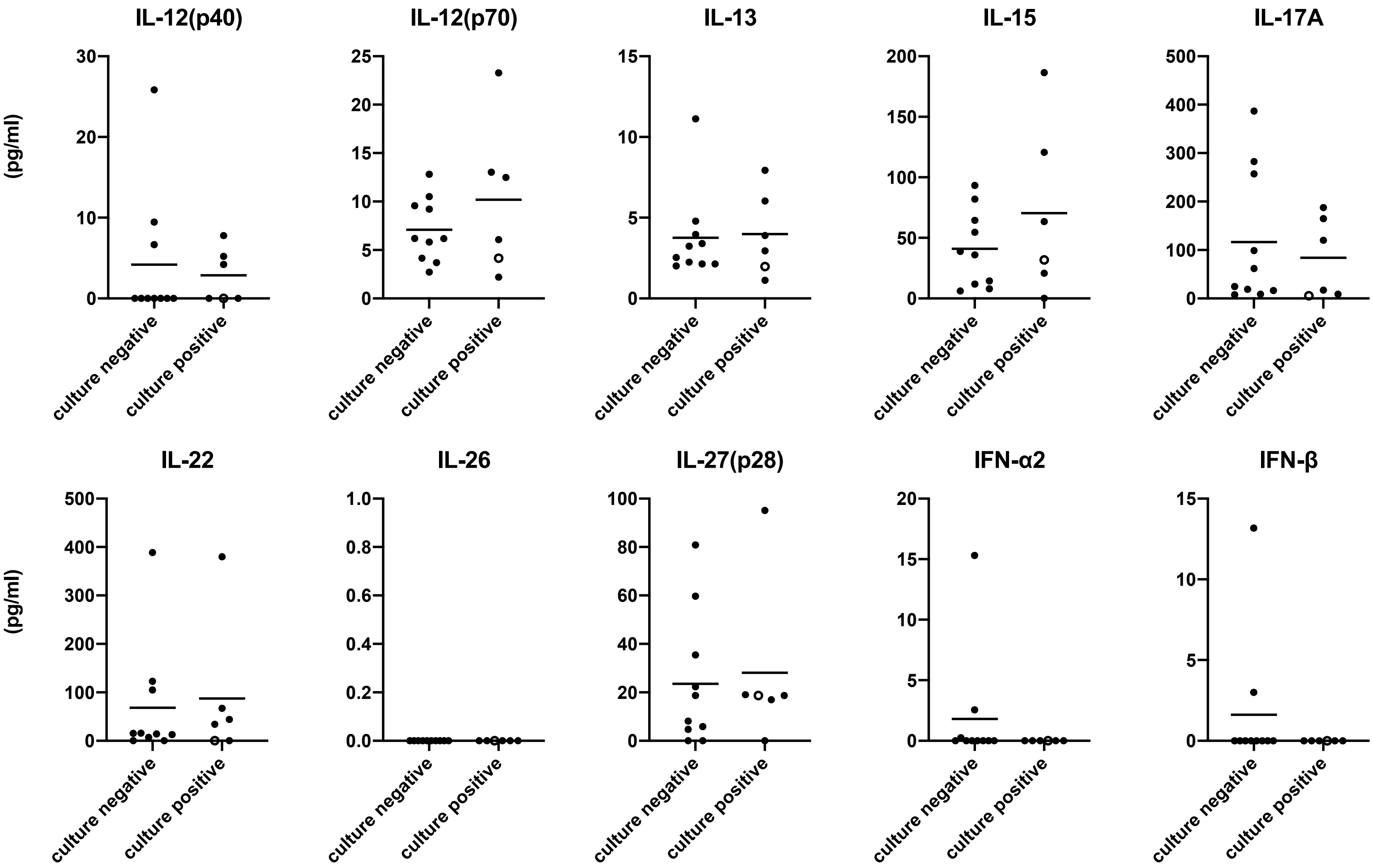

IL-12(p40)
IL-12(p70)
IL-13
IL-15
IL-17A
IL-22
IL-26
IL-27(p28)
IFN-α2
IFN-β
(pg/ml)
culture negative
culture positive

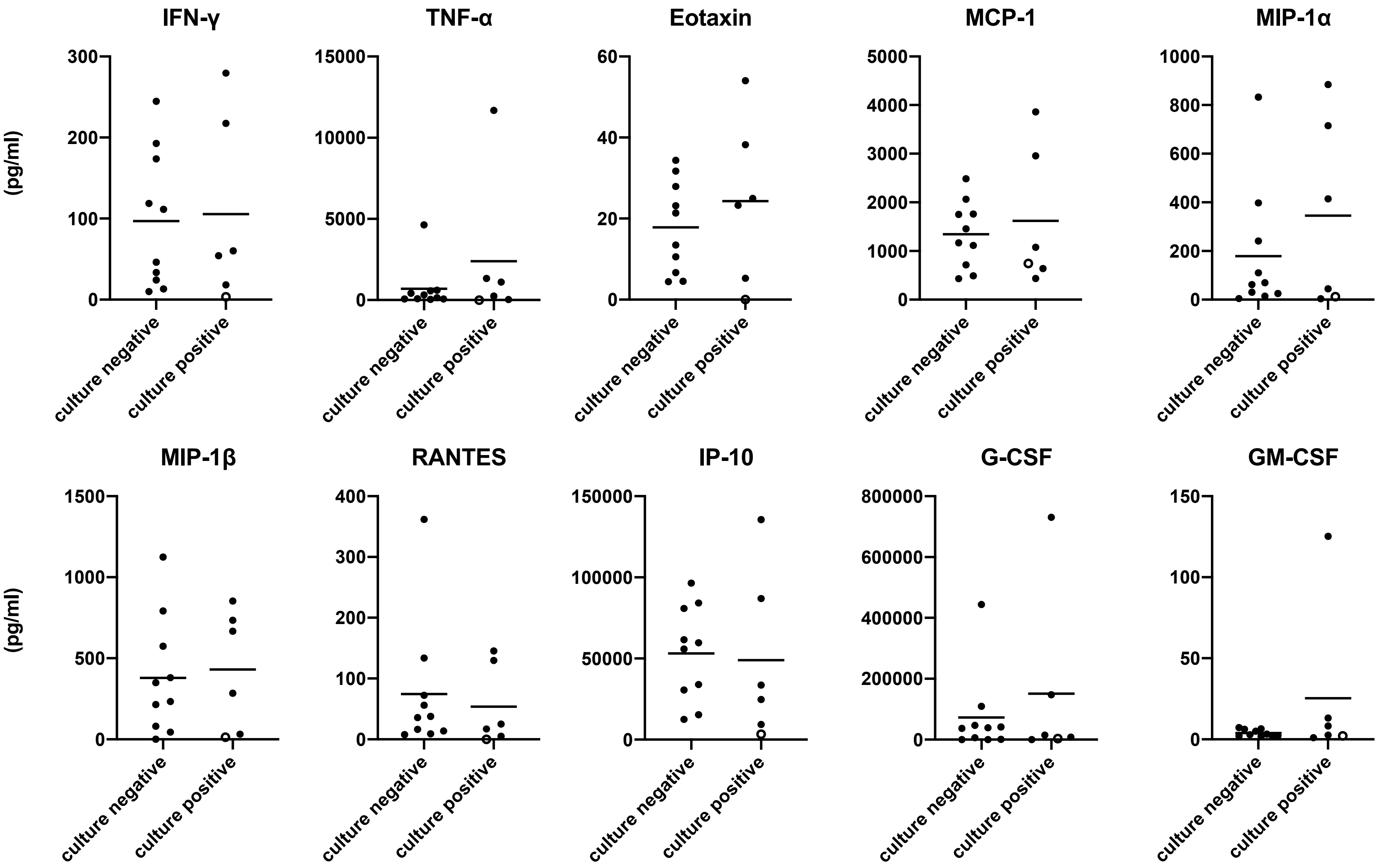

IFN-γ
TNF-α
Eotaxin
MCP-1
MIP-1α
MIP-1β
RANTES
IP-10
G-CSF
GM-CSF
(pg/ml)
(pg/ml)
culture negative
culture positive
0
100
200
300
5000
10000
15000
20
40
60
1000
2000
3000
4000
400
600
800
500
1500
150000
50000
100000
200000
400000
600000
800000
50
150

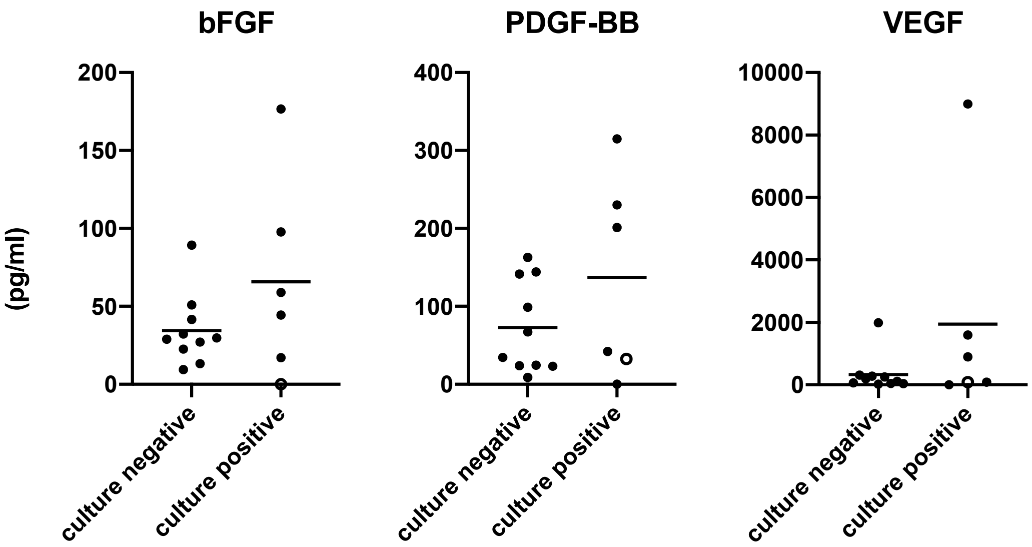
bFGF
PDGF-BB
VEGF
(pg/ml)
200
150
100
50
0
400
300
200
100
0
10000
8000
6000
4000
2000
0
culture negative
culture positive

Supplementary Table S1. Patients' clinical details at the time of sample collection

Diseases	ERM	IOL	sarcoidosis	ARN	BE
Timing of the vitrectomy from the onset of symptoms (months, mean ± SD)	unknown	10.82 ± 9.57	18.23 ± 22.64	0.62 ± 0.35	0.20 ± 0.20
Anterior inflammation score (mean ± SD)	0 ± 0	0.42 ± 0.64	0.9 ± 0.85	1.65 ± 0.80	3.56 ± 0.63
Vitreous opacity score (mean ± SD)	0 ± 0	2.08 ± 0.95	1.9 ± 0.93	2 ± 0.71	3.63 ± 0.62
Retinitis score (mean ± SD)	0 ± 0	0.54 ± 0.97	1 ± 0.85	2.46 ± 0.78	2.31 ± 1.14
Presence of hypopyon at the vitrectomy (cases, %)	0 (0%)	0 (0%)	0 (0%)	0 (0%)	13 (81.25%)
Systemic steroid use (cases, %)	0 (0%)	2 (15.38%)	5 (33.33%)	12 (92.31%)	1 (6.25%)
Steroid subconjunctival injection before surgery within 2 months (cases, %)	0 (0%)	0 (0%)	3 (20%)	1 (7.69%)	0 (0%)
Topical steroid eye drops (cases, %)	0 (0%)	4 (30.77%)	14 (93.33%)	13 (100%)	7 (43.75%)
Systemic antibiotic/ antiviral agents (cases, %)	0 (0%)	0 (0%)	0 (0%)	12 (92.31%)	7 (43.75%)
Pseudophakic eyes at the vitrectomy (cases, %)	3 (20%)	6 (46.15%)	5 (33.33%)	0 (0%)	11 (68.75%)

ARN, acute retinal necrosis; BE, bacterial endophthalmitis; ERM, idiopathic epiretinal membrane; IOL, intraocular lymphoma; SD, standard deviation

Supplementary Table S2. Statistical data of immune mediators in vitreous humor of patients with ERM and other 4 uveitis diseases using Kruskal-Wallis test

Cytokines	p-values
IL-1β	**<.0001**
IL-1ra	**<.0001**
IL-2	**<.0001**
IL-4	**<.0001**
IL-5	**<.0001**
IL-6	**<.0001**
IL-7	**0.0001**
IL-8	**<.0001**
IL-9	**<.0001**
IL-10	**<.0001**
IL-12(p40)	0.4725
IL-12(p70)	**0.0001**
IL-13	**0.0003**
IL-15	**0.0175**
IL-17A	**<.0001**
IL-22	**<.0001**
IL-26	**0.0138**
IL-27(p28)	**0.0006**
IFN-α2	**<.0001**
IFN-β	0.101
IFN-γ	**<.0001**
TNF-α	**<.0001**
Eotaxin	**<.0001**
MCP-1	**<.0001**

MIP-1α	**<.0001**
MIP-1β	**<.0001**
RANTES	**<.0001**
IP-10	**<.0001**
G-CSF	**<.0001**
GM-CSF	**<.0001**
bFGF	**<.0001**
PDGF-BB	**0.001**
VEGF	**<.0001**

Significant differences ($P < 0.05$) are in bold.
